# CDK5 Inhibition Abrogates TNBC Stem‐Cell Property and Enhances Anti‐PD‐1 Therapy

**DOI:** 10.1002/advs.202001417

**Published:** 2020-10-15

**Authors:** Yuncheng Bei, Nan Cheng, Ting Chen, Yuxin Shu, Ye Yang, Nanfei Yang, Xinyu Zhou, Baorui Liu, Jia Wei, Qin Liu, Wei Zheng, Wenlong Zhang, Huifang Su, Wei‐Guo Zhu, Jianguo Ji, Pingping Shen

**Affiliations:** ^1^ State Key Laboratory of Pharmaceutical Biotechnology and The Comprehensive Cancer Center Nanjing Drum Tower Hospital The Affiliated Hospital of Nanjing University Medical School Nanjing University Nanjing 210046 P. R. China; ^2^ Laura and Isaac Perlmutter Cancer Center New York University Langone Medical Center New York NY USA; ^3^ State Key Laboratory Cultivation Base for TCM Quality and Efficacy Nanjing University of Chinese Medicine Nanjing 210023 P. R. China; ^4^ State Key Laboratory of Protein and Plant Gene Research College of Life Sciences Peking University Beijing 100871 P. R. China; ^5^ The Comprehensive Cancer Center Nanjing Drum Tower Hospital The Affiliated Hospital of Nanjing University Medical School Nanjing 210008 P. R. China; ^6^ Guangdong Key Laboratory of Genome Instability and Human Disease Shenzhen University Carson Cancer Center Department of Biochemistry and Molecular Biology Shenzhen University School of Medicine Shenzhen 518060 P. R. China

**Keywords:** cancer stem cells, CD44 variants, CDK5, immune checkpoint blockade, PPAR*γ* phosphorylation, triple‐negative breast cancer, tumor microenvironment

## Abstract

Triple‐negative breast cancer (TNBC) is the most aggressive subtype of breast cancer, in which the higher frequency of cancer stem cells (CSCs) correlates with the poor clinical outcome. An aberrant activation of CDK5 is found to associate with TNBC progression closely. CDK5 mediates PPAR*γ* phosphorylation at its Ser 273, which induces CD44 isoform switching from CD44s to CD44v, resulting in an increase of stemness of TNBC cells. Blocking CDK5/pho‐PPAR*γ* significantly reduces CD44v+ BCSCs population in tumor tissues, thus abrogating metastatic progression in TNBC mouse model. Strikingly, diminishing stemness transformation reverses immunosuppressive microenvironment and enhances anti‐PD‐1 therapeutic efficacy on TNBC. Mechanistically, CDK5 switches the E3 ubiquitin ligase activity of PPAR*γ* and directly protects ESRP1 from a ubiquitin‐dependent proteolysis. This finding firstly indicates that CDK5 blockade can be a potent strategy to diminish stemness transformation and increase the response to PD‐1 blockade in TNBC therapy.

## Graphical abstract

Cancer stem cells (CSCs) promote triple‐negative breast cancer (TNBC) metastasis and therapeutic resistance, including immunotherapy. Aberrant activation of CDK5 promotes TNBC progression by triggering stemness transformation. Mechanistically, CDK5 switches the non‐canonical E3 ubiquitin ligase activity of PPARγ and protects ESRP1 from a ubiquitin‐dependent proteolysis, which controls CD44v generation. Pharmacological interruption of CDK5 reverses immunosuppressive tumor microenvironment, and enhances anti‐PD‐1 curative effects.

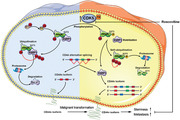

## 1 Introduction

Triple‐negative breast cancer (TNBC) is a highly malignant subtype of breast cancer (BC), which is defined by the lack of expression of the estrogen receptor (ER), progesterone receptor (PR), and human epidermal growth factor receptor 2 (HER2). Up to 95% of TNBCs are classified as invasive mammary carcinomas, characterized by marked heterogeneity, aggressive distant metastasis, and relatively higher proportion of breast cancer stem cells (BCSCs) compared with other BC subtypes.^[^ ^1^, ^2^ ^]^ Despite the current standard cure modalities, such as surgery, chemotherapy, and radiotherapy, TNBC patients have a high rate of relapse and drug resistance, and BCSCs are deemed to be one of the most important causes.^[^ ^3^, ^4^ ^]^ TNBC patients with CSCs markers mostly exhibit a worse prognosis.^[^ ^5^ ^]^ Eliminating CSCs is potentially beneficial to tumor therapy efficacy and durable tumor remission.^[^ ^6^ ^]^

Cyclin‐dependent kinase 5 (CDK5) is a non‐canonical cyclin‐dependent kinase that has been well‐characterized for the development of the central nervous system by regulating neuronal development and differentiation.^[^ ^7^ ^]^ Its activation is primarily dependent on binding to the non‐cyclin activators CDK5R1 (p35) and CDK5R2 (p39), thereby phosphorylating a list of substrates, such as retinoblastoma (RB) protein, signal transducer and activator of transcription 3 (STAT3), and peroxisome proliferator‐activated receptor‐gamma (PPAR*γ*).^[^ ^8^, ^9^ ^]^ With these critical roles, CDK5 is intrinsically involved in many cellular events, including proliferation, adhesion, migration, and apoptosis, and has been revealed to serve as a key node in many physiological and pathological activities especially in cancer.^[^ ^8^ ^]^ A number of studies have shown that aberrant CDK5 activity contributes to tumor progression by promoting angiogenesis, metastasis, and immune evasion.^[^ ^8^, ^10^ ^]^ In particular, as to the role of CDK5 in BC, it has been indicated that the inhibition and disruption of CDK5 attenuate BC growth via orchestrating a migration–proliferation dichotomy.^[^ ^11^ ^]^ CDK5 possesses the dominant effect in controlling the BC cell cycle in a CDK2‐independent manner.^[^ ^12^ ^]^ Additionally, inhibiting CDK5 can promote the sensitivities of BC cells to multi‐kinds of chemotherapeutic drugs, such as 5‐fluorouracil, 6‐thioguanine, and paclitaxel, significantly.^[^ ^13^ ^]^ These provocative findings imply that CDK5 might, therefore, be another key molecular target in the CDK family for cancer treatment after the identification of CDK4 and CDK6 as effective and stable drug targets. Indeed, a set of new drugs to inhibit CDK5 activity are being developed in several clinical trials (NCT01333423, NCT01580228, and NCT01676753). In this context, further deciphering of the mechanistic underpinning for CDK5 deregulation is urgently needed, and this may help develop new targeted molecular strategies and novel clinical combinations.

Recently, the successful treatment of some patients with malignancies using immune checkpoint blockade (ICB) antibodies, such as those directed against the programmed cell death protein 1 and its ligand (PD‐1/PD‐L1) axis and cytotoxic T lymphocyte‐associated antigen 4, has demonstrated unprecedented clinical resolution, which has truly brought new hope to refractory TNBC.^[^ ^14^ ^]^ Indeed, the proof‐of‐principle studies with ICB in advanced‐stage TNBC have yielded promising results compared with the outcome from other BC subtypes.^[^ ^15^ ^]^ However, emerging data from preclinical and clinical trials suggest limited success with ICB monotherapy in TNBC. Thus, several investigations and clinical trials, evaluating the integration of ICB into the adjuvant combinatorial settings, are underway, and some of them have shown promising success in TNBC patients.^[^ ^2^, ^16^ ^]^ Recently, increasing studies have revealed that the populations of tumor cells with CSC traits are elevated after immunotherapy, which is essential for adaptive resistance and tumor recurrence.^[^ ^17^ ^]^ Although, Mukherjee et al. have documented a novel link between CDK5 and glioma CSCs,^[^ ^10^ ^]^ the mechanism underlying CDK5‐mediated TNBC stemness remains unclear and it is still unknown whether CDK5 blockade efficiently enhances immunotherapy in TNBC. Here, we sought to investigate a rationale for combining CDK5 inhibition with ICB to achieve TNBC remission, while exploring the corresponding underlying mechanism by which CDK5 promotes stemness transformation in TNBC progression.

## 2 Results

### 2.1 Clinical Relevance of CDK5 Activation in TNBC Progression

**Figure 1:**
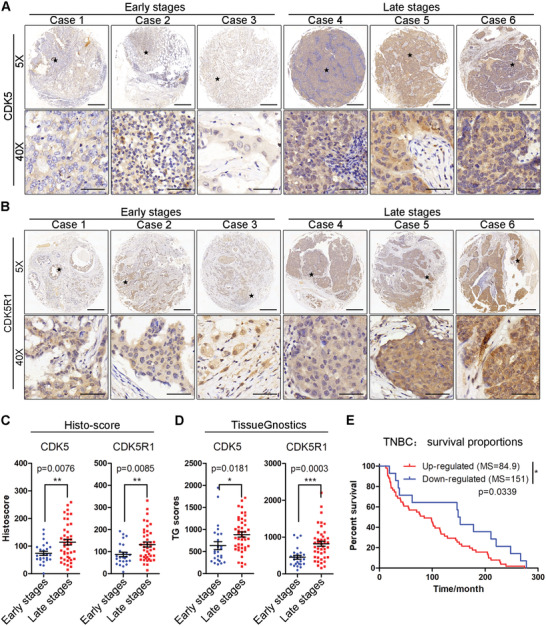
Aberrant expressions of CDK5 and CDK5R1 clinically correlate with TNBC progression. Immunohistochemistry (IHC) detection of A) CDK5 and B) CDK5R1 expression in tissue microarrays, classified into early‐stage (I/II, *n* = 26) and late‐stage (III/IV, *n* = 44). Scale bars, 500 × 10^−6^ m for (5 ×) and 50 × 10^−6^ m for (40 ×). C) Histoscores for CDK5 and CDK5R1 staining in different stages. D) Expression of CDK5 and CDK5R1 in each clinical specimen was quantified using TissueGnostics GmbH FACS‐like Tissue Cytometry (TissueFAXS Plus). E) Kaplan–Meier analysis of the overall survival (OS) of TNBC patients according to CDK5 expression: upregulated (red line, *n* = 54, median OS 84.9 months), and downregulated (blue line, N = 24, OS 151 months). Data represent the analysis of *n* patient specimens per group, mean ± SEM. *p < 0.05, **p < 0.01, ***p < 0.001.

To understand the correlation between CDK5 activation and each subtype of BC, we assessed the mRNA expression levels of CDK5, CDK5R1, and CDK5R2 using online analysis in the cBioPortal database (METABRIC, Nature, 2012 & Nature Communications, 2016).^[^ ^18^ ^]^ This database is composed of 1699 BC cases including 679 Luminal A, 461 Luminal B, 220 HER2‐positive (HER2+), 299 TNBC, and 140 normal breast tissues. As shown in Figure S1A–C in the Supporting Information, CDK5 and CDK5R1, but not CDK5R2, were significantly elevated in TNBC at the mRNA level compared with other subtypes as well as normal breast tissues, indicating an essential role of CDK5 in TNBC. Consistently, the same results were seen in other cohorts of clinical samples (Figure S1E,F, Supporting Information). Kaplan–Meier curves were measured to analyze whether the survival time of each BC subtype was associated with the expression of CDK5. The results showed that among TNBC patients, those with higher expression of CDK5 showed a shorter overall survival time (**Figure** **1**E). In other BC subtypes, there was no significant correlation between CDK5 expression and overall survival (Figure S1D, Supporting Information). Comprehensive consideration based on these results placed our focus on TNBC for further study. To clarify the importance of CDK5 in TNBC, we prepared a tissue microarray (TMA) in a set of 70 human TNBCs, including 26 cases in early stages (stage I/II) and 44 cases in late stages (III/IV), and analyzed the expression of CDK5 and CDK5R1 using immunohistochemistry (IHC) (Figure 1A,B). The immunostained TMA slides were then quantified using the Histoscore and TissueGnostics software. It was notable that CDK5 expression, in line with CDK5R1, in the late stages was significantly higher than that in the early stages (Figure 1C,D). Additionally, CDK5 and CDK5R1 were overexpressed in tumor tissues of patients with metastasis (Figure S1G, Supporting Information). All these clinical data indicate the possibility that aberrant CDK5 activity plays a vital role in the development of human TNBC.

### 2.2 CDK5 Promotes TNBC Metastasis via Controlling Stemness Transformation

**Figure 2:**
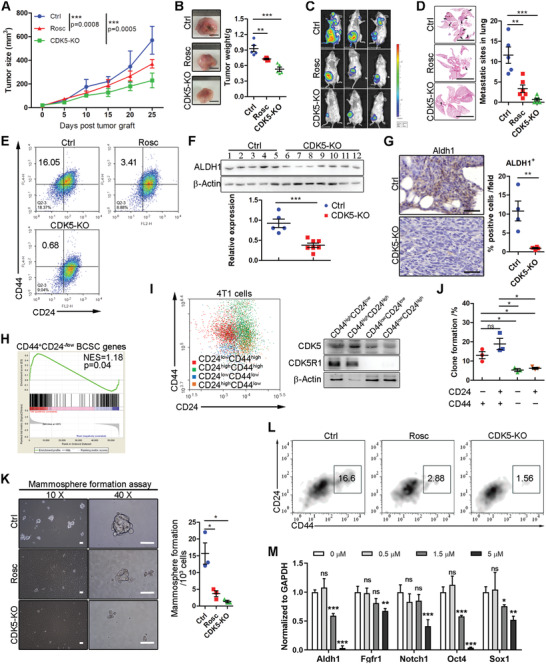
CDK5 blockade inhibits metastasis and stemness in the TNBC mouse model. A) The growth of orthotopic 4T1 tumors in mice with different treatments as indicated (*n* = 6 for each group). B) Left, representative images of tumor tissues isolated from mice described in A). Right, mean tumor weights are shown. C) Representative bioluminescence images of 4T1‐bearing mice with indicated treatments. D) Left, representative pictures of hematoxylin and eosin‐stained (H&E) sections of lung tissues. Right, quantification of pulmonary metastatic nodules. E) Representative flow cytometric analysis of CD44^+^CD24^−/low^ BCSC population in 4T1 tumors. F) Immunoblot analysis of CSCs marker, ALDH1, expression in tumor tissues with or without Rosc treatment. *β*‐Actin was analyzed as a loading control. The ALDH1 expression level was quantified using ImageJ. G) Representative IHC images for ALDH1 expression in 4T1 tumor tissues with indicated treatments. The ratios of ALDH1‐positive cells in each field were quantified. H) Gene set enrichment analysis of alterations in BCSC signature genes after CDK5 inhibition. I) Immunoblot analysis of CDK5 and CDK5R1 expression in each subtype of 4T1 cells as indicated. J) Colony formation analysis of each subtype of 4T1 cells. K) Mammosphere formation in 4T1 cells treated as indicated. Left, a representative image of mammospheres. Right, quantification of mammospheres, counted using a microscope with size ≥ 50 µm. Scale bars = 50 µm. L) Representative flow cytometric analysis of CD44^high^CD24^−/low^ BCSC population in mammospheres. M) mRNA level of BCSC‐related signature genes (Aldh1, Fgfr1, Notch1, Oct4, and Sox1). Data represent mean ± SEM. The experiments were repeated at least twice to observe concordant statistical significance. *p < 0.05, **p < 0.01, ***p < 0.001.

To investigate the exact role of CDK5 in TNBC progression, we performed a series of in vivo and in vitro experiments. First, we constructed a CDK5 knockout cell line (CDK5‐KO). The efficient sgRNA was screened and selected to deplete endogenous CDK5 (Figure S2A,B, Supporting Information). Then, we established an orthotopic mouse model by using CDK5‐KO cells or parental 4T1 cells.^[^ ^19^ ^]^ Additionally, the mice bearing the parental 4T1 cells were also challenged with roscovitine (Rosc), a potent CDK5 inhibitor (2 mg kg^−1^ 5 days^−1^). The results showed that multiple tumor nodules that had metastasized to the lung surface were observed in the control group, but none was seen in the CDK5‐KO group (**Figure** **2**C,D), while tumor volume and tumor weight were slightly reduced after CDK5 depletion (Figure 2A,B). These results were further supported by Rosc treatment (Figure 2A–D). Using extracellular matrix composition analysis, we found that the collagen deposition and the downregulated expression of MMP‐2/9 were mediated by CDK5 depletion or Rosc treatment, respectively, indicating a favorable extracellular matrix for metastasis (Figure S2D, Supporting Information). These combined data suggest that CDK5 is critical for TNBC metastasis.

Next, we examined how CDK5 promotes TNBC metastasis. Flow cytometric analysis indicated that CDK5 depletion abrogated the formation of the CD44^high^CD24^low^ BCSC population significantly (Figure 2E). Concomitantly, immunoblotting and immunohistochemical staining documented that the BCSCs marker gene, aldehyde dehydrogenase 1 (ALDH1), was downregulated after CDK5 depletion (Figure 2F,G). Similar results were observed in Rosc‐treated mice (Figure 2E), implying the involvement of CDK5 in TNBC stemness transformation. Epithelial‐mesenchymal transition (EMT) is one of the major characters of CSCs promoting CSC expansion and anchorage‐independent growth at the invasive fronts of tumors, thus facilitating tumor metastasis.^[^ ^20^ ^]^ Next, the EMT phenotype of tumors was determined by immunohistochemical assessment. As shown in Figure S2C in the Supporting Information, a more epithelial‐like phenotype was induced in both CDK5‐KO and Rosc‐treated tumors, defined by increased E‐cadherin and reduced vimentin expression. Collectively, CDK5 promoted TNBC metastasis majorly by enhancing stemness transformation.

To further corroborate the function of CDK5 in mediating TNBC stemness, firstly, we conducted an expression profiling after Rosc treatment in 4T1 cells. Gene set enrichment analysis (GSEA) revealed that several stemness‐related pathways, such as Hedgehog, Hippo, JAK/STAT, PI3K/AKT/mTOR, and RAS/ERK/MAPK,^[^ ^4^ ^]^ were downregulated in Rosc‐treated cells (Figure S2E, Supporting Information). Moreover, the gene signature of CD44^+^CD24^−/low^ BCSCs, proposed by Creighton et al.,^[^ ^21^ ^]^ was governed by CDK5 (Figure 2H). These results suggest that the capacity of CDK5 to positively control gene expression is associated with stem‐like traits. Next, we determined the expression pattern of CDK5 and CDK5R1 in heterogeneous 4T1 cells. According to identified tumorigenicity markers, CD44 and CD24, we isolated the BCSC (CD44^high^CD24^low^) population together with other subpopulations using flow cytometry (Figure 2I). As previously observed,^[^ ^22^ ^]^ the CD44‐positive population, including BCSC and CD44^high^CD24^high^ populations, showed elevated tumorigenicity compared with the other two (Figure 2J). Interestingly, in these cells with high tumorigenicity, CDK5 and CDK5R1 expressions were both amplified (Figure 2I). Furthermore, we performed a mammosphere formation assay (MFA) to evaluate the self‐renewal capacity of tumor cells in vitro. As shown in Figure 2K, CDK5 knockout or Rosc treatment significantly prevented mammosphere formation. Flow cytometric analysis discovered an enriched population of CD44^high^CD24^low^ in the mammosphere, which was strikingly attenuated by Rosc treatment or CDK5 depletion (Figure 2N). Additionally, a number of BCSC marker genes, were downregulated upon Rosc treatment, in a dosage‐dependent manner (Figure 2M). Collectively, these data demonstrate the pivotal role of CDK5 in TNBC stemness transformation during metastatic progression.

### 2.3 CDK5 Controls CD44v Isoform Switching Responsible for TNBC Stemness

**Figure 3:**
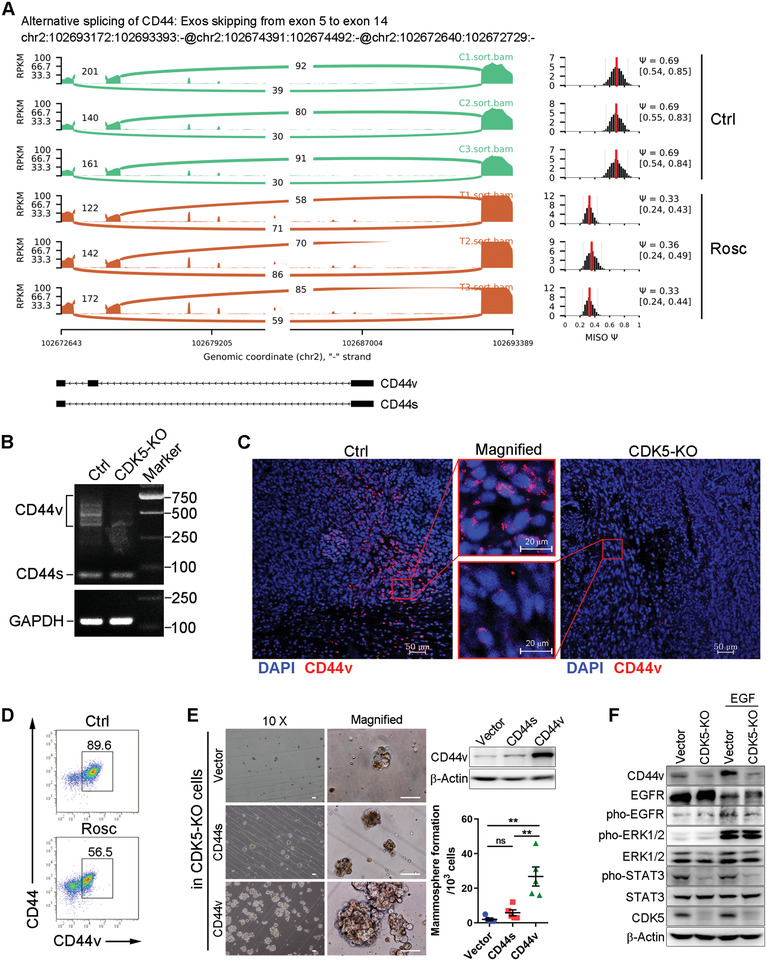
CDK5 controls CD44v isoform switching promoting TNBC stemness. A) Sashimi plot showing a large number of reads spanning CD44 exons 5 and 14 junctions in Rosc‐treated cells. B) RT‐PCR detection of CD44 variant fragment expression. C) Representative immunofluorescence microscopy images for the analysis of CD44v expression (red) in 4T1‐bearing mice with/without Rosc treatment. Nuclei were counterstained with DAPI. Scale bars, 50 and 20 µm for the magnified field. D) Flow cytometric analysis of CD44v expression in 4T1 tumors. E) Mammosphere formation assay detecting self‐renewal capacity. The genetic recovery experiment was conducted by overexpressing CD44s/CD44v in CDK5‐KO cells as indicated. F) Immunoblotting analysis of the activation of CD44v downstream EGFR signaling pathway. Data represent mean ± SD. *p < 0.05, **p < 0.01, ***p < 0.001.

The cell surface protein CD44 is a major representative marker for BCSCs. Recently, several studies have discovered that CD44 is of great importance to fulfilling the special properties of CSCs, such as self‐renewal and tumor microenvironment (TME) construction.^[^ ^23^ ^]^ As mentioned above, the aberrant expression of CDK5 was dominantly enriched in CD44‐positive 4T1 cells, indicating a close link between CDK5 and CD44. Therefore, we speculated that CD44 might be involved in CDK5‐induced TNBC stemness transformation. As expected, the cassette exon alternative splicing events in CD44 were impacted by CDK5 interruption according to the Sashimi plot (**Figure** **3**A). However, no difference was evident in CD44 expression, identified using flow cytometric analysis (Figure S3A, Supporting Information). This data implied the great capacity of CDK5 in modulating CD44 alternative splicing. To further explain this phenomenon, the CD44 exon‐specific qPCR was utilized as described previously.^[^ ^24^ ^]^ The forward and reverse primers were designed to target the exon 5 and 16 of CD44, for amplifying CD44 variant fragments. The result showed that CDK5 depletion induced a switch in CD44 mRNA splicing that led to the generation of the CD44s mRNA rather than CD44v mRNA (Figure 3D). To evaluate the endogenous and exogenous CD44v expression, we generated an anti‐CD44v antibody to specifically recognize CD44v rather than CD44s. The specificity of the anti‐CD44v antibody was evaluated using immunoblotting and flow cytometry (Figure S3B,C, Supporting Information). Then, we determined CD44v expression in tumors formed by CDK5‐KO cells using immunofluorescent staining with an anti‐CD44v antibody. We noticed that CDK5 depletion significantly decreased CD44v expression in vivo, and a similar result was observed in the Rosc‐treated tumors (Figure 3C,D). Together, CD44v isoform switching is particularly controlled by CDK5.

To substantiate the conclusion that CDK5 induces TNBC stemness transformation by triggering CD44v isoform switching, we performed a gain‐of‐function assay in CDK5‐KO cells by overexpressing CD44s or CD44v. It was noteworthy that in the cells with CD44v overexpression, mammosphere formation was strikingly evoked even under CDK5 depletion (Figure 3E). In contrast, the forced expression of CD44s, as well as the control vector, was unable to restore the self‐renewal capacity of 4T1 cells suppressed by CDK5‐KO (overcome CDK5‐KO‐induced suppression of the self‐renewal capacity of 4T1 cells) (Figure 3E). Moreover, some certain stemness‐related signaling pathways,^[^ ^25^, ^26^ ^]^ spontaneously activated by CD44v overexpression in 4T1 cells (Figure S3D, Supporting Information), were markedly restrained subjecting them to CDK5 depletion (Figure 3F). Additionally, consistent with CDK5, CD44v was majorly overexpressed in TNBC tumors compared with the other subtypes of BC (Figure S3E, Supporting Information). Our combined results demonstrate that CDK5 controls CD44v isoform switching to elicit TNBC stemness.

### 2.4 PPAR*γ* Phosphorylation is Necessary for CDK5‐Induced CD44v Class Switching

Next, we searched for the CDK5 downstream molecules that drive the alternative splicing of CD44. Given that CDK5 belongs to the proline‐directed protein kinase, we screened potential substrates of CDK5 via phosphoproteome profiling. As shown in Figure S4A in the Supporting Information, PPAR*γ* was enriched in 4T1 cells with CDK5 overexpression. Referring to the findings that CDK5 mediates the phosphorylation of PPAR*γ* at the Ser273 site in adipocytes, proposed by Choi et al.,^[^ ^27^ ^]^ our preliminary results provided a notable hint that PPAR*γ* was possibly involved in CDK5‐induced CD44v class switching.

**Figure 4:**
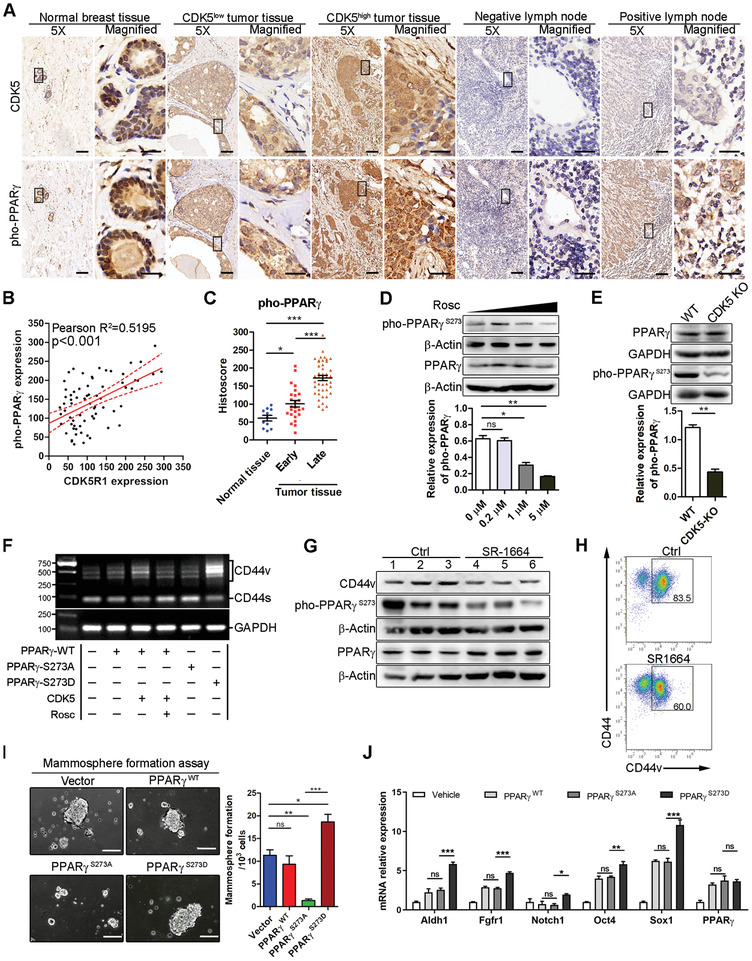
PPAR*γ* phosphorylation is necessary for CDK5‐induced CD44v class switching. A) Representative IHC images for CDK5 and pho‐PPAR*γ* expression in tissue microarrays, including normal breast and tumor tissues and negative and positive lymph nodes. Typical staining in the box region was magnified and shown in the adjacent columns. Scale bars, 20 × 10^−6^ m for (5×) and 50 × 10^−6^ m for (magnified). B) Correlation of CDK5 expression and phosphorylation level of PPAR*γ* (*n* = 70, Pearson's correlation coefficient *R* and *p*‐value are shown). C) Histoscores of pho‐PPAR*γ* IHC staining. Normal breast tissue (*n* = 11); stage 1 (*n* = 26) and stage 2 (*n* = 44). D,E) Immunoblotting analyzing the expression of phosphorylation of PPAR*γ* at the Ser273 site in 4T1 cells after CDK5 interruption. D) Rosc treatment; E) CDK5 knockout. The levels of pho‐PPAR*γ* were semi‐quantified according to the gray value calculated using ImageJ (bottom panel). F) CD44v‐targeted RT‐PCR detection. Examination of CD44v expression using G) immunoblotting and H) flow cytometry in 4T1 tumors after SR‐1664 treatment. I) Mammosphere formation assay (MFA) detecting the self‐renewal capacity of transgenic 4T1 cells as indicated. J) qPCR detection of stemness‐related gene expression. Data represent mean ± SD. *p < 0.05, **p < 0.01, ***p < 0.001.

To assess this possibility, we established a series of in vitro and in vivo assays. Firstly, we investigated whether CDK5 triggered PPAR*γ* phosphorylation in human TNBC specimens. To this end, we performed the consecutive TMA and examined CDK5 expression and PPAR*γ* phosphorylation at the Ser273 site using a specific antibody.^[^ ^28^ ^]^ As shown in **Figure** **4**A, in two consecutive TMA slides, phosphorylated PPAR*γ* (pho‐PPAR*γ*) and CDK5 were closely co‐localized in the tumor tissues. Additionally, the pho‐PPAR*γ* level was much higher in CDK5‐high specimens than that in CDK5‐low specimens. Pearson's correlation analysis showed a significantly positive correlation between pho‐PPAR*γ* and CDK5 in the tumor tissues (Pearson *r* = 0.5195, *p* < 0.001) (Figure 4B). Moreover, similar to CDK5, pho‐PPAR*γ* expression was mainly detected in the tumor tissues, especially in those of the late‐stage (Figure 4C), as well as the lymph node with metastasis (Figure 4A). Consistent with our observation in the clinical specimens, blocking CDK5 by pharmacologic inhibition with Rosc or CDK5 depletion significantly abrogated the phosphorylation of PPAR*γ* at the Ser273 site in vitro and in vivo (Figure 4D,E; Figure S4B,C, Supporting Information). These data, therefore, signify that CDK5 augments the phosphorylation of PPAR*γ* in TNBC.

We then investigated whether pho‐PPAR*γ* could control CD44v isoform switching. To do so, we produced 4T1 cells by overexpressing wild type or mutated PPAR*γ*, as indicated in Figure S4D in the Supporting Information. The serine at site 273 of PPAR*γ* was mutated into aspartic acid (PPAR*γ*‐S273D) or alanine (PPAR*γ*‐S273A) to mimic the hyper‐ or non‐phosphorylated form, respectively. Based on the CD44v‐specific PCR assay, which is similar to CDK5, PPAR*γ*‐S273D enhanced CD44v isoform generation (Figure 4F). However, the opposite result was seen in the PPAR*γ*‐S273A cells (Figure 4F), indicating the important contribution of pho‐PPAR*γ* to CD44v isoform switching. In support of these data, we treated 4T1‐bearing mice with SR‐1664, a non‐agonist ligand that specifically blocks CDK5‐mediated PPAR*γ* phosphorylation at the Ser273 site.^[^ ^29^ ^]^ In this setting, reducing pho‐PPAR*γ* resulted in a significant decrease in CD44v (Figure 4G,H). Finally, to further explore whether pho‐PPAR*γ* promoted TNBC stemness, we investigated the stemness‐related property in vitro and in vivo. Analogous to CDK5, overexpressing pho‐PPAR*γ* enhanced mammosphere formation (Figure 4I). Moreover, the BCSC marker genes increased significantly in PPAR*γ*‐S273D cells (Figure 4J). In contrast, overexpressing PPAR*γ*‐S273A abrogated stemness transformation in vitro and in vivo (Figure 4I; Figure S4F, Supporting Information), which resulted in the marked suppression of tumor growth and pulmonary metastasis (Figure S4E,G, Supporting Information). Therefore, these results demonstrate that CDK5‐induced pho‐PPAR*γ* drives CD44v isoform switching during TNBC stemness transformation.

### 2.5 Interruption of CDK5/pho‐PPAR*γ* Axis Induces Fate Alteration of TNBC Stem Cell

To elucidate the mechanism underlying the effect of CD44v isoform switching, we examined the mode of action of pho‐PPAR*γ* mediated by CDK5 in triggering TNBC stemness transformation. Since PPAR*γ* is the transcriptional factor, we first examined the association between PPAR*γ* transcriptional activity and its promotive role in stemness transformation. To this end, a series of identified small molecules were used for CDK5/pho‐PPAR*γ* axis interruption (Figure S5B, Supporting Information); meanwhile, the self‐renewal capacity of 4T1 cells was parallelly analyzed using MFA (Figure S5A, Supporting Information). As shown in Figure S5D in the Supporting Information, the fact that PPAR*γ*‐triggered self‐renewal capacity was closely associated with pho‐PPAR*γ* level (Figure S5C, Supporting Information), rather than its transcriptional activity. These data implied that CDK5/PPAR*γ* axis‐triggered TNBC cell stemness transformation was independent of PPAR*γ* transcriptional activity.

**Figure 5:**
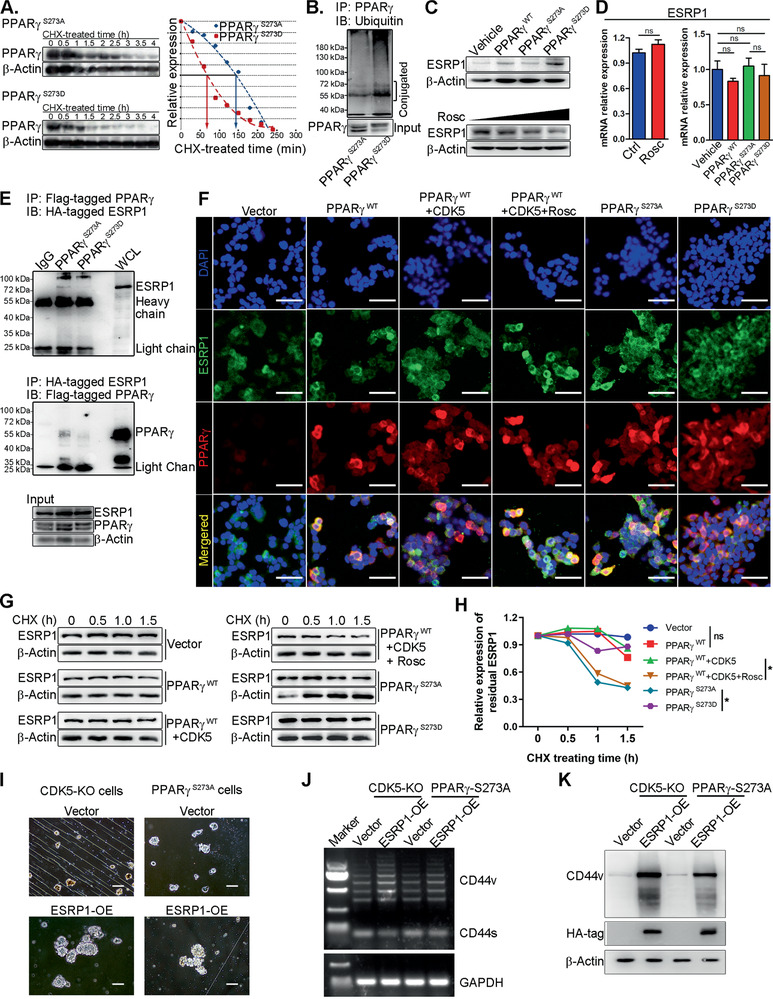
Interruption of the CDK5/pho‐PPAR*γ* axis induces fate alteration of TNBC stem cells. A) 4T1 cells were overexpressed with PPAR*γ*‐S273A and PPAR*γ*‐S273D. Cycloheximide treatment revealed the half‐life of PPAR*γ* with a different mutation. B) The transgenic cells described in (A) were pre‐incubated with MG132 (10 × 10^−6^ m) for 2 h, then the cell lysates were subjected to ubiquitination detection. C) Immunoblotting analysis of ESPR1 expression in transgenic 4T1 cells as indicated (top) or after incubation with Rosc in different concentrations (bottom). D) RT‐qPCR detection of the expression of ESRP1 at the mRNA level. E) Co‐immunoprecipitation (co‐IP) assay analyzing the interaction between PPAR*γ* and ESRP1 (top). The interaction was further confirmed using reverse co‐IP (bottom). F) Representative fluorescence confocal microscopy images analyzing the co‐localization (yellow) between PPAR*γ* (red) and ESRP1 (green) in different treatments as indicated. Nuclei are stained with DAPI (blue). Scale bars, 50 µm. G,H) In 4T1 cells with different treatments as indicated, cycloheximide treatment examined the half‐life of ESRP1. G) Immunoblotting detected the residual of ESRP1 at the protein level and H) consequently quantified it by normalizing the intensity of the actin band. I) Transient overexpression of ESRP1 in CDK5‐KO and PPAR*γ*‐S273A cells. MFA detected self‐renewal capacity. J) CD44v‐targeted RT‐PCR detection examined CD44v generation. K) Immunoblotting detected CD44v expression in the indicated cells after ESRP1 transient overexpression. Data represent mean ± SD. *p < 0.05, **p < 0.01, ***p < 0.001.

Next, we searched for the potential PPAR*γ*‐interacting proteins to elucidate the rational mechanism by applying an anti‐Flag affinity purification mass spectrometry (MS) assay. To preliminarily evaluate the binding strength, the list of candidate proteins was subjected to online docking analysis tools, such as ZDOCK, PatchDOCK, and FireDOCK.^[^ ^30^ ^]^ Notably, epithelial splicing regulatory protein 1 (ESRP1) showed the strongest binding strength with PPAR*γ*, as shown in Figure S5E in the Supporting Information. For further substantiation of the interaction between PPAR*γ* and ESRP1, co‐immunoprecipitation assays were elaborately performed. As shown in **Figure** **5**E, ESRP1 coimmunoprecipitated with PPAR*γ* in PPAR*γ*‐S273A cells. A similar result was observed in a reverse co‐immunoprecipitation assay (Figure 5E). However, in the PPAR*γ*‐S273D cells, phosphomimetic mutation significantly antagonized the interaction between PPAR*γ* and ESRP1 (Figure 5E). Furthermore, fluorescence confocal microscopy imaging indicated that the interruption of the CDK5/pho‐PPAR*γ* axis largely abolished the physical interaction between PPAR*γ* and ESRP1 (Figure 5F). These data not only proved the exact interaction between PPAR*γ* and ESRP1 but also demonstrated the pivotal role of CDK5‐mediated phosphorylation of PPAR*γ* in its interaction with ESRP1.

Next, we further evaluated the biological consequences of ESRP1 after direct binding. To this end, we examined the expression of ESRP1 after the interruption of the CDK5/pho‐PPAR*γ* axis in 4T1 cells. Notably, blocking the CDK5/pho‐PPAR*γ* axis significantly decreased ESRP1 expression at the protein level (Figure 5C) even though there were no obvious alterations in ESRP1 at the mRNA expression level (Figure 5D), indicating a non‐canonical role of PPAR*γ* in protein stability regulation.^[^ ^31^ ^]^ Given that ubiquitin‐mediated proteolysis is a key process to control protein stability post‐translationally, we speculated that PPAR*γ* could mediate ESRP1 ubiquitination. To assess this hypothesis, we performed a ubiquitination assay as described previously.^[^ ^32^ ^]^ In the PPAR*γ*‐S273A cells, non‐phosphorylated PPAR*γ* elicited the ubiquitination of ESRP1 (Figure 5F). In contrast, the ubiquitination of ESRP1 was significantly hampered after the induction of the CDK5/pho‐PPAR*γ* axis (Figure 5F). Furthermore, cycloheximide treatment revealed that the ESRP1 protein turnover rate was significantly impacted by pho‐PPAR*γ* (Figure 5G,H). Considering that ESRP1 is proposed as a master proto‐oncogenic mRNA splicing regulator responsible for CD44 alternative splicing in breast cancer,^[^ ^33^, ^34^ ^]^ we did further genetic rescue experiments. As expected, the transient expression of ESRP1 restored the self‐renewal capacity of CDK5‐KO cells and PPAR*γ*‐S273A cells (Figure 5I), by inducing CD44v generation (Figure 5J,K). Based on these results, we concluded that the interruption of the CDK5‐/pho‐PPAR*γ* axis was necessary and sufficient to alter the fate of TNBC stem cells.

Hou et al.’s findings that PPAR*γ* possesses the potential E3 ligase‐like activity^[^ ^31^ ^]^ enlightened us to pursue the exact enzymatic merit of pho‐PPAR*γ* further. Catalyzing self‐ubiquitination is a typical feature of most E3 ligases, which is widely used for ligase identification.^[^ ^35^ ^]^ Similarly, in vitro ubiquitination assay revealed that pho‐PPAR*γ* enhanced self‐ubiquitination, and promoted self‐degradation (Figure 5B). However, impairment of CDK5‐mediated phosphorylation strengthened the binding to ESRP1 (Figure 5E,F), which protected PPAR*γ* from self‐destruction (Figure 5A). These data indicated that CDK5 switched the E3 ubiquitin ligase activity of PPAR*γ* and directly protected ESRP1 from ubiquitin‐dependent proteolysis, triggering CD44v generation, thus resulting in TNBC stemness transformation.

### 2.6 Pharmacological Inhibition of Stemness Transformation of Tumor Cells Enhances Anti‐PD‐1 Therapeutic Efficacy

Despite the importance of CSCs in tumor development,^[^ ^36^ ^]^ the regulation of CSCs in tumor immune microenvironment construction has not been extensively studied. In this study, the CDK5 interruption‐mediated decline of stemness transformation of TNBC cells significantly remodeled the TME toward an anti‐tumoral state (Figure S6B–D, Supporting Information). Especially, CD8+ cytotoxic T lymphocytes (CTLs) were notably infiltrated in tumors after CDK5 inhibition (Figure S6C, Supporting Information), which strongly predicted the response to ICB.

**Figure 6:**
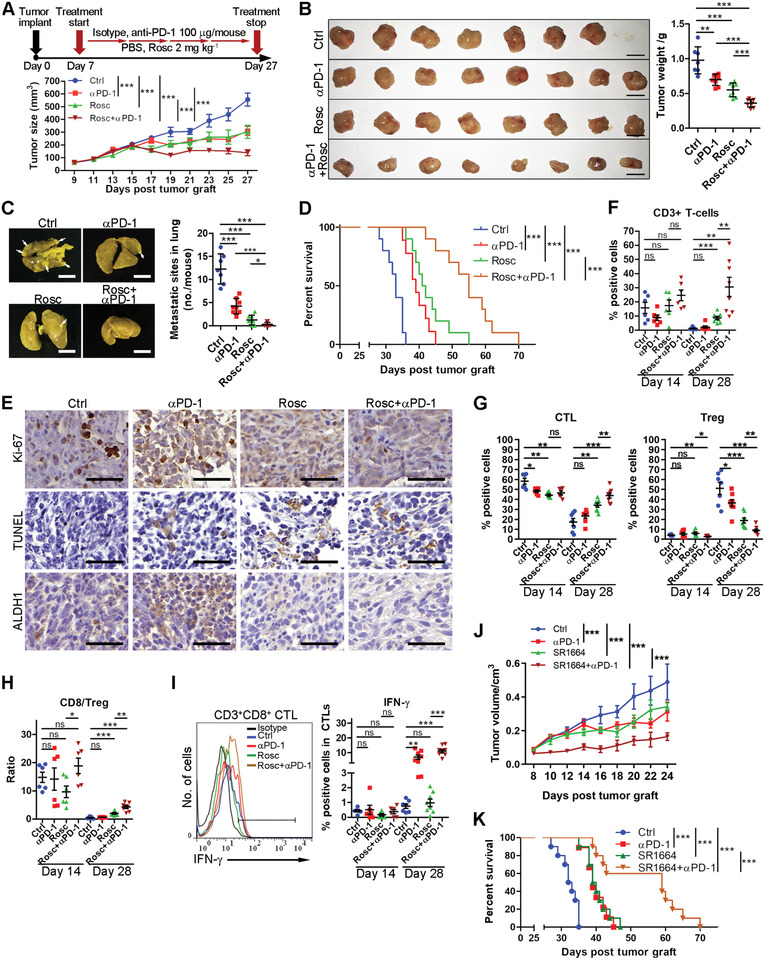
Pharmacological inhibition of the stemness transformation of tumor cells enhances anti‐PD‐1 therapeutic efficacy. A) Therapy regimen (top panel) and mean tumor volume of orthotopic 4T1‐bearing mice treated with vehicle or Rosc (2 mg kg^−1^), combinational treatment with isotype and anti‐PD‐1 antibodies (100 mg per mouse) for every five days (*n* = 8, 1 dead in the control group, bottom panel). B) Representative photographs of 4T1 tumor tissues isolated at the time of termination of the experiment (scale bars, 1 cm). Tumor weights are presented in the right panel. C) Representative photographs of lung metastasis are shown in the left panel, and quantification of metastatic sites is shown in the right panel. D) Kaplan–Meier survival analysis for the overall survival of mice. E) Representative IHC images of Ki‐67 and ALDH1 in 4T1 tumors with indicated treatments. Apoptosis was detected using the TUNEL assay. Scale bars, 50 µm. F) Quantification of CD3+ T‐cell, G, left) CTL, and G, right) Treg populations and H) the ratio of CTLs/Tregs in 4T1 tumors at days 14 and 28 post tumor graft (*n* = 6) using flow cytometry. I) Representative flow cytometry images and quantification of IFN‐*γ* expression in CD8^+^ TILs (*n* = 6). J,K) Examination of the therapeutic effects of the combined treatment with anti‐PD‐1 and SR‐1664 in 4T1 tumors. J) mean tumor volumes were recorded at the indicated times. K) The overall survival of each group was analyzed using the Kaplan–Meier survival analysis. Data represent mean ± SD. *p < 0.05, **p < 0.01, ***p < 0.001.

**Figure 7:**
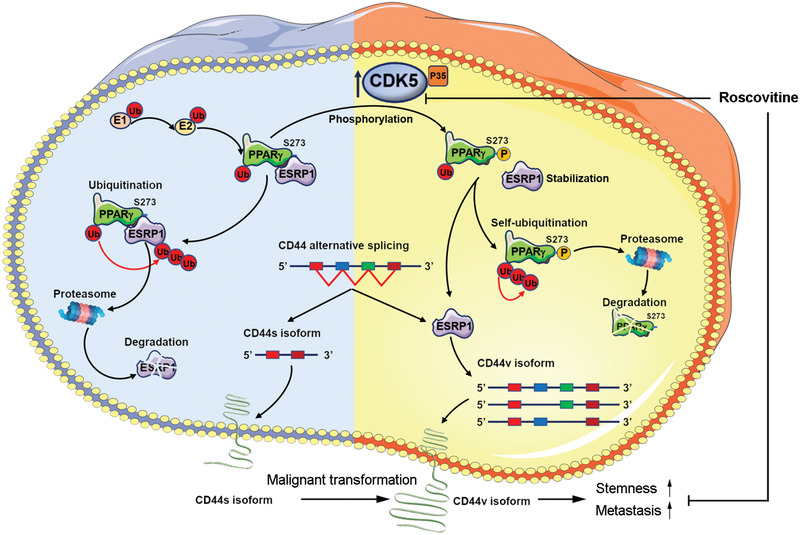
Schematic summary. The mechanisms underlying TNBC stemness transformation. CDK5 expression increases during TNBC cell stemness transformation, and CDK5 phosphorylates PPAR*γ* at Ser273 and modulate its E3 ligase activity. This alteration stabilizes ESRP1 and promotes CD44v isoform generation. CD44v isoform switching causes stemness transformation and metastasis. CDK5 inhibition abrogates CD44v isoform switching and enhances anti‐PD‐1 therapy. (up arrow for increase, down arrow for decrease)

To estimate whether inhibiting tumor cell stemness transformation improves the efficacy of ICB on TNBC, we established the anti‐PD‐1 therapy resistance mouse model as described previously.^[^ ^19^ ^]^ Then, the mice were injected with Rosc (2 mg kg^−1^) or anti‐PD‐1 antibody (100 µg per mouse), individually or in combination, on days 7, 12, 17, and 22 (Figure 7A, top). In accordance with a previous finding,^[^ ^19^ ^]^ individual treatment of anti‐PD‐1 exhibited limited therapeutic effects on TNBC. However, combination treatment with Rosc and anti‐PD‐1 strongly inhibited tumor growth and metastatic progression (**Figure** **6**A–C), while the treated mice showed a slight increase in body weight (Figure S6E, Supporting Information). Survival analysis confirmed the superiority of the combination treatment (Figure 6D). To further evaluate the synergistic therapeutic effects of the combination treatment, the proliferation, apoptosis, and stemness transformation of the tumor cells were examined using IHC. As shown in Figure 6E, Ki67 (a proliferation marker) decreased and apoptosis was induced in the tumor tissues after CDK5 inhibition, and the combination exerted additive effects. Importantly, enhanced stemness transformation in tumor tissues after anti‐PD‐1 treatment was diminished markedly by CDK5 inhibition (Figure 6E). These data suggested that abrogating CDK5‐mediated stemness transformation induced a decline in the malignant extent of the tumor cells, and favored anti‐PD‐1 therapeutic effects in TNBC mouse model.

Next, we assessed the effects of combined treatment on the TME. To dynamically capture the characteristics of the TME, tumor tissues from each group were collected on day 14 (early stage) and 28 (late‐stage) post‐transplantation. Prepared single‐cell suspensions were subsequently subjected to multicolor flow cytometric analysis. The gating strategy is shown in Figure S6A in the Supporting Information. In late‐stage tumor tissues, the ratio of anti‐tumoral immune cells, such as CTLs (Figure 6G) and M1‐prone macrophages (Figure S6G, Supporting Information), decreased significantly, while pro‐tumoral immune cells, including M2‐prone macrophages (Figure S6G, Supporting Information), MDSCs (Figure S6J, Supporting Information), and regulatory T cells (Treg) (Figure 6G), increased highly. These results indicated an obvious shift from a supportive microenvironment to the immunosuppressive one during TNBC development, as previously reported.^[^ ^19^ ^]^ Notably, we observed that the percentage of tumor‐infiltrating lymphocytes (TILs) increased significantly in tumors with combined treatment (Figure S6F, Supporting Information). High infiltration of TILs is a clinical biomarker, indicating the beneficial responses of ICB therapy.^[^ ^37^ ^]^ Furthermore, immunophenotyping tumors with combined treatment using flow cytometry showed, in addition to an increase in the overall percentage of CD3+ cells (Figure 6F), an increase in CD3+CD8+ CTLs, interferon‐*γ* (IFN‐*γ*)‐expression CD8+ T‐cells, and M1‐prone macrophages, (Figure 6G–I). By contrast, there was a decreasing trend in regulatory T‐cells (Tregs) and M2‐prone macrophages (Figure 6G; Figure S6G, Supporting Information). No significant changes were observed in infiltrated dendritic cells and MDSCs (Figure S6I,J, Supporting Information). Moreover, the ratios of CTLs/Treg and M1/M2 were tremendously elevated, indicating the tipping of the immune balance (Figure 6H; Figure S6H, Supporting Information). These data indicated that attenuating CDK5‐mediated stemness transformation modified the constitution of the immune cells in the TNBC TME, which improved the efficacy of anti‐PD‐1 therapy. Since pho‐PPAR*γ* was proven to augment TNBC cell stemness transformation as well, we further investigated whether pharmacological inhibition of pho‐PPAR*γ* similarly enhanced anti‐PD‐1 therapy. To this end, the curative effects of SR‐1664 as an individual application or in combination with anti‐PD‐1 were comprehensively examined. As expected, the combined treatment with SR‐1664 and anti‐PD‐1 significantly suppressed tumor growth, and markedly extended overall survival in the TNBC mouse model, compared with the individual treatment with SR‐1664 or anti‐PD‐1 (Figure 6J,K).

Conclusively, pharmacologically diminishing tumor cells stemness transformation by targeting the CDK5/pho‐PPAR*γ* axis causes an obvious immunophenotyping shift, thus augmenting the efficacy of anti‐PD‐1 therapy in TNBC.

## 3 Discussion

In the current study, we provided novel insights into the mechanisms underlying TNBC cell stemness transformation and demonstrated that CDK5 plays an essential role during TNBC cell stemness transformation in vitro and in vivo (**Figure** **7**). In the CDK family, the most well‐known members to date are CDK4 and CDK6 because they are the fundamental drivers of the cell cycle and are responsible for the carcinogenesis and progression in many types of cancers.^[^ ^38^ ^]^ Notably, a class of selective CDK4/6 inhibitors, including palbociclib, ribociclib, and abemaciclib, has shown significant clinical activities across a range of malignancies.^[^ ^38^ ^]^ Unlike CDK4/6, CDK5 is a non‐stereotypical CDK member and its activation is majorly dependent on binding to non‐cyclin activators CDK5R1 and CDK5R2.^[^ ^8^ ^]^ The multifunctional properties of CDK5, specifically in regulating carcinogenesis and cancer progression have been overlooked for many years. Until recently, several studies have provided increasing evidence at the gene, mRNA, and protein levels unveiling the role of CDK5 in human cancers, including BC, non‐small cell lung cancers (NSCLCs), and brain cancers.^[^ ^8^ ^]^ It has been proven that CDK5 inhibition contributes to eliminate the progression of certain cancers and overcome the resistance to anticancer therapies efficiently.^[^ ^8^ ^]^ These investigations indicated the possibility that CDK5 could be an intriguing drug target. Indeed, several pharmacological CDK5 inhibitors have been undergoing preclinical/clinical trials for possible anticancer applications and are yielding promising responses.^[^ ^39^ ^]^ In the current study, we first found the existence of CDK5 activation in tumor tissues of TNBC patients at different stages and then established the primary positive correlation between aberrant CDK5 activation and TNBC progression. By applying multiple experimental approaches, we demonstrated that CDK5 is indeed critically required for TNBC cell stemness transformation, which has a strong association with enhanced metastasis and the constitution of an immunosuppressive environment. CDK5 blockade not only results in profound metastasis abrogation but also promote anti‐tumor immunity.

There is a consensus recognition that CDK5 triggers a cascade of events to promote tumor progression by phosphorylating its specific substrates on serine and/or threonine residues.^[^ ^8^ ^]^ Actually, the knowledge gained about the exact interpretation of the linkage between CDK5 and its substrates in BC cells has helped in understanding the neoplastic roles of CDK5 and further broadened the spectrum for CDK5‐targeted treatment in BC.^[^ ^13^, ^40^ ^]^ Here, on account of the detailed context‐based quantitative analysis of specimens from TNBC patients, we observed a close correlation between CDK5 activation and PPAR*γ* phosphorylation at the Ser273 site. Thus, we postulated that TNBC‐related CDK5 activation might lead to PPAR*γ* phosphorylation. Indeed, further comprehensive analysis suggested that there was an eventual considerable kinase‐substrate relationship between CDK5 and PPAR*γ* in the context of TNBC. Retrospectively, Bruce's group first found in adipocytes an event where CDK5 induces PPAR*γ* phosphorylation at the Ser273 site.^[^ ^29^ ^]^ This finding indicates a novel modality of post‐translational modification of PPAR*γ* creating a new setting to further explore the biological actions of PPAR*γ*.^[^ ^27^ ^]^ Enlightened by this idea, we performed a series of tests, including focused genetic screening and subsequent mechanistic studies, to elucidate the precise significance of PPAR*γ* phosphorylation mediated by CDK5 in TNBC progression. First, unraveling the interaction between PPAR*γ* and ESRP1 revealed that PPAR*γ* exhibited E3 ligase activity.^[^ ^31^ ^]^ Strikingly, CDK5 switched this non‐canonical biological activity of PPAR*γ* and directly protected ESRP1 from ubiquitin‐dependent proteolysis. All these molecular events occurred during BC cell stemness transformation inducing CD44v generation, which is the canonical biomarker of CSCs and a valuable indicator of poor prognosis.^[^ ^34^, ^41^ ^]^ In summary, these data implied that CDK5 surely control TNBC cell stemness transformation. With this, we tuned the action of the CDK5/pho‐PPAR*γ* axis to analyze the exact role of this node in programming stemness. The results demonstrated that CDK5/pho‐PPAR*γ* axis activation is positively responsible for the malignant progression of TNBC in both human specimens and experimental models. The pharmacological inhibition or genetic interruption of CDK5/pho‐PPAR*γ* truly diminished tumor development in the TNBC mouse model. Collectively, these results strongly suggest that the CDK5/pho‐PPAR*γ* axis exists in the context of TNBC stemness. This molecular axis is a pivotal driver of the aggressive development of TNBC.

Developmental preclinical studies have demonstrated the tremendously promotive anti‐tumoral efficacy of antibody‐based strategies against CD44v in multiple human cancers.^[^ ^26^, ^34^, ^41^, ^42^ ^]^ According to the restricted CD44v expression in cancer cells and the conclusive association between CD44v isoform and poor prognosis, CD44v has been regarded as a valuable diagnostic and therapeutic target in certain human cancers, including gastric, colorectal, hepatocellular, and breast carcinoma^[^ ^43^ ^]^ On this basis, a number of humanized antibodies against CD44v are undergoing clinical trials, and have shown promising curative effects (NCT01358903 and NCT01641250).^[^ ^44^ ^]^ However, there remains the challenge of the clinical application of antibody‐based treatments. For example, the immunogenic accumulation of antibodies in nontumor areas limited the clinical use of bivatuzumab (a humanized monoclonal antibody against CD44v6).^[^ ^44^ ^]^ Additionally, the highly complicated and context‐dependent expression of CD44v isoforms substantiates the necessity and urgency to generate adequate new antibodies with high affinity against each isoform.^[^ ^43^ ^]^ Although some other approaches appear to interrupt CD44v in addition to a humanized antibody,^[^ ^45^ ^]^ the fundamental questions about CD44v‐targeted treatment are still unsolved. Therefore, the good knowledge of the upstream regulatory mechanism of CD44 alternative splicing is helpful for us to understand how CD44v promotes malignant transformation. From a broader perspective, the pivotal molecular nodes identified in the network responsible for CD44v generation prefer to provide potential druggable targets for further pharmaceutical development. Of note, the novel regulatory pathway centering on CDK5 for controlling CD44v generation was originally shown in this study. More importantly, using the small molecules, including interrupting CDK5 activity and inhibiting PPAR*γ* phosphorylation at 273, have proven to efficiently block CD44v isoform switching, which exhibits promotive antitumoral effects. The regulatory mechanism developed by us opens a new avenue to device a novel strategy against CD44v by targeting CDK5 for TNBC therapy.

CSC has been reported to create and maintain populations of tumor‐associated immune cells, such as TAMs and Tregs, to build up an immunosuppressive niche for immune evasion.^[^ ^46^ ^]^ Although the mechanism that regulates the interaction of CSCs with the immune system has not yet been well defined, we speculated that successfully targeting CSCs proactively remodel their microenvironment, thus enhancing immunotherapeutic efficacy. To confirm this hypothesis, we established and comprehensively verified the therapeutic effects of combinatorial treatments with CDK5 inhibitor and anti‐PD‐1 antibody in the TNBC mouse model. As expected, diminishing stemness transformation by targeting CDK5 profoundly promoted anti‐PD‐1 curative effects. Moreover, we observed the underappreciated function of CDK5 in enhancing TILs infiltration, indicating a narrow association between CSCs and T‐cell infiltration in tumor tissues.^[^ ^47^ ^]^ In conclusion, these results bring us new hope that targeting CDK5 may sensitize TNBC and other immunologically “cold” cancer types to immunotherapy.

## 4 Conclusion

This study unearthed the vital function of CDK5 in inducing the stemness transformation of TNBC cells and promoting malignant progression. Concerning the molecular mechanism, CDK5 was uncovered to enhance CD44 alternative splicing from CD44s to CD44v isoform in driving TNBC cell stemness transformation. On one hand, enhanced CD44v generation was proven to provoke the self‐renewal capacity of TNBC cells. On the other hand, CD44v‐positive CSCs would also facilitate immunosuppressive microenvironment construction. The latter mechanism was verified in BC cells during stemness transformation. This novel mechanism not only highlighted the essential role of CDK5 in regulating PPAR*γ* E3 ubiquitin ligase activity but also opened new avenues for devising a novel strategy against CD44v by targeting CDK5 for TNBC therapy. Additionally, CDK5 interruption with anti‐PD‐1 therapy displayed a striking therapeutic efficacy in TNBC. With this pilot study, more efforts are needed to further address CDK5‐targeted pharmaceutical development and translational issues in the future.

## 5 Experimental Section

##### Cell Culture

The 4T1 (GDC0294) murine mammary cell lines were obtained from China Center for Type Culture Collection (Wuhan, China), and maintained in RPMI 1640 media supplemented with 1% penicillin–streptomycin and 10% fetal bovine serum (Gibco). All cells were cultured at 37 °C with 5% CO_2_.The cell lines were mycoplasma tested.

##### CRISPR‐CAS9 Knockout

Online sgRNA designer were used (http://crispr.mit.edu/, donated by Zhang Feng) to target the common exons for all mice, CDK5 isoforms were synthesized as follows: 5′‐ ACAGCCGCAACGTGCTACAT ‐3′, and cloned to lentiCRISPRv2 (one vector system) plasmids from Zhang lab. Scramble sequence: 5′‐ GGCTCCAACCGTCCAGAATA ‐3′.

##### Site‐Directed Mutagenesis

The PPAR*γ*‐S273A, S273D mutants were generated using Plenti‐PPAR*γ* plasmid as a template by polymerase chain reaction with a Muta‐Direct Site‐Directed Mutagenesis Kit (SBS Genetech Co., Ltd., China). The primers are listed as follows: PPAR*γ*‐S273A forward:5′‐CAGGAAAGACAACGGACAAAGCACCATTTGTCATCTACGAC‐3′, PPAR*γ*‐S273A reverse: 5′‐GTCGTAGATGACAAATGGTGCTTTGTCCGTTGTCTT TCCTG‐3′; PPAR*γ*‐S273D forward: 5′‐CAGGAAAGACAACGGACAAAGAACCA TTTGTCATCTACGAC‐3′, PPAR*γ*‐S273D reverse: 5′‐GTCGTAGATGACAAATGG TTCTTTGTCCGTTGTCTTTCCTG‐3′. The changes in the nucleotide bases are underlined. The site‐specific mutations and absence of any spurious mutations were confirmed by DNA sequencing at Genscript Company.

##### CD44 Variant Specific Monoclonal Antibody Preparation

The purified His‐CD44v (V2‐V10) fusion protein was used to immunize female BALB/c mice according to the conventional procedure.^[^ ^48^ ^]^ Briefly, the splenic B cells were purified and fused with mouse myeloma Sp2/0 cells by PEG. Positive hybridomas were tested by ELISA and Western blot analysis, and were cloned by limiting dilution process. The hybridoma cell lines that secreted antibody with high titers were massively cultured for hybridoma injection. Seven days later, ascites were collected and centrifuged at 12 000 r min^−1^ for 30 min to obtain the supernatant. The anti‐CD44v monoclonal antibody was purified by using protein A/G affinity chromatography. Specificity and titration were examined by Western blot, IHC, and immunofluorescence microscopy in cells or tissues.

##### Animal Study

Female BALB/c mice (6–8 weeks old) were purchased from the Model Animal Research Center of the Nanjing University, Nanjing, China and bred in the animal facilities under specific pathogen‐free conditions. All mouse procedures and experiments for this study were approved by Institutional Animal Care and Use Committee, Nanjing University (Accreditation No. 2017‐S09‐72). For 4T1 model, 2 × 10^5^ cells suspended in RPMI, 1640 were injected into the inguinal mammary fat pads of syngeneic, immunocompetent BALB/c mice. Treatments were given as single agents or in combinations with the following regimen for each drug. The CDK5 inhibitor Roscovitine was administered by intraperitoneal (i.p.) injection every 5 days at 2 mg kg^−1^. Anti‐PD‐1 antibody (100 µg per mouse, clone RPM1‐14, Bio X cell) was intravenously (i.v.) injected every 5 days. Treatment was initiated when tumors became palpable on day 7 ending on day 27 post tumor implant. Tumors were measured every second day with the vernier caliper, and the volume (0.5 × length × width × width) was calculated.

##### Human Triple‐Negative Breast Cancer Tissue Microarray

This study was approved by the Institutional Review Board at The Nanjing University. All human tissues were obtained from Chinese PLA General Hospital, Beijing. All patients provided written informed consent in accordance with the declaration of Helsinki before enrolling in the study. The protocol in this study was approved by the institutional review board at the Chinese PLA General Hospital, Beijing. No commercial sponsor was involved in this study. Human tissue microarrays from 70 cases of human TNBC (including 26 cases in stage 1 and 44 cases in stage 2 according to the TNM staging system) were analyzed by immunohistochemistry staining using CDK5 (1:50) and CDK5R1 (1:50) antibodies. The immunostained slides were quantified by Histoscore and TissueGnostics software.

##### Analysis of Genes Expression from Human Databases

Gene expression data of human BC from The Cancer Genome Atlas (TCGA) database (https://cancergenome.nih.gov/) was used to analyze selected genes expression during BC progression in different subtypes of BC. Only patients who had been classified into different BC subtypes (including luminal A, luminal B, HER2+ and TNBC) according to PAM50 were taken into account . Normalized expression data and *z*‐scores for mRNA expression data were downloaded from cBioPortal (www.cbioportal.org).

##### Gene Set Enrichment Analysis

GSEA was performed by using preranked tool as described previously.^[^ ^49^ ^]^ For each conditional gene‐requirement experiment, a ranked list and a conditional essential gene set were created. Each ranked list was assessed against each gene sets. Enrichment score (ES) was then calculated to reflect the degree to which a set gene was overrepresented. The ES was then normalized for each gene set to account for the size of the set, as normalized enrichment score (NES). False discovery rate was used to generate the *p* values for significant enrichment.

##### Phosphoproteome

Phosphoproteome was performed as previously described.^[^ ^50^ ^]^ Briefly, proteins were extracted from each cell lines. Then protein extracts were incubated with PTMScan Motif specific antibody following manufacturer's recommendations. The capture proteins were then eluted with dilute acid and analyzed by LC–MS/MS.

##### TMT‐Based Quantitative Proteomics

The whole proteins were dissolved in 2% sodium dodecyl sulfate (SDS), 100 × 10^−3^ m tris, pH 8.5, and were subjected to tryptic digestion. 100 µg digested peptides were labeled with TMT isobaric labeling reagents (ThermoFisher Scientific) in buffer with a hydrous acetonitrile for 1 h at room temperature, and 8 µL of 5% hydroxylamine was added to quench the reaction. Equivalent labeled peptides were combined and desalted on a C18 Extraction Disk Cartridge (3M Empore, Agilent Technologies). The labeled peptides were subjected to online strong anion exchange (SCX)‐reversed phase liquid chromatography‐tandem MS.

##### Mammoshpere Formation Assay

6‐Well plates were pre‐coated with 1 mL poly (2‐hydroxyethyl methacrylate) (pHEMA) (12 g pHEMA was dissolved in 1 L 95% ethanol; one needs to keep stirring overnight to dissolve pHEMA completely). The pre‐coated plates were then placed at 37 °C for 48–72 h. Cultured cells were trypsinized and resuspended in DMEM/F12 media (Gibco). The number of viable cells were calculated, and then the cells were seeded as 1 × 10^4^ per well in 2 mL DMEM/F12 media supplemented with insulin, transferrin, selenium (ITS) and 20 ng mL^−1^ EGF (Sigma Aldrich). Cells were cultured at 37 °C with 5% CO_2_. Spheres greater than 50 µm were counted 12–14 days later.

##### Immunohistochemistry, Immunofluorescence, RT‐qPCR, and Western Blot

Immunohistochemistry, immunofluorescence, RT‐qPCR and Western blot were performed according to standard protocols as previously described.^[^ ^51^ ^]^ Primers are listed in Table S1 in the Supporting Information. All antibody information is listed in Table S2 in the Supporting Information.

##### Immunoprecipitation

For the immunoprecipitation assays, whole‐cell extracts were prepared after transfection, incubated overnight with the appropriate antibodies, and subsequently incubated with protein A/G beads (Beyotime) for 2 h. The beads were then washed five times with low‐salt lysis buffer, and the immunoprecipitates were eluted from beads in 1× SDS loading buffer (Beyotime) and boiled for 10 min, and then resolved by SDS‐PAGE. Proteins were transferred to PVDF membranes (Bio‐Rad) and incubated with the appropriate antibodies. The LumiGlo Chemiluminescent Substrate System (Thermo Fisher Scientific) was used for protein detection.

##### Flow Cytometry

All cells were pre‐incubated with FcR blocking antibody (FcR Blocking Reagent, Miltenyi Biotec) for 15 min at 4 °C at concentration of 1 µg per 1 × 10^6^ cells in 100 µL PBS. Fluorescent antibodies and isotype antibodies were consequently added at the indicated concentration, and cells were incubated for a further 30 min at 4 °C. Then cells were washed twice with ice‐cold PBS. Flow cytometry analysis was performed with the BD FACS calibur and data were analyzed with FlowJo software.

##### Statistical Analysis

All statistical analyses were performed by GraphPad Prism 5 (GraphPad). TCGA RNAseq_V2 gene expression data was downloaded from cBioPortal website. Differences between two groups were calculated by Student's *t*‐test. Multiple comparisons between two populations were conducted by multiple *t*‐tests with type 1 error correction. Differences among multiple groups were calculated by one‐ or two‐way ANOVA. Differences in survival were calculated by Log‐rank Mantel‐Cox test. Differences between tumor growth curve were determined by repeated measures two‐way ANOVA. Significance was set at *p* values of or below 0.05. For all figures, *p < 0.05, **p < 0.01, ***p < 0.001. Unless noted in the figure legend, all data are shown as mean ± SEM. Generally, all experiments were carried out with *n* ≥ 3 biological replicates.

## Conflict of Interest

The authors declare no conflict of interest.

## Author Contributions

P.S. supervised the project and wrote the manuscript; Y.B. designed the study, performed most experiments, and bioinformatics analysis; N.C., T.C., and Y.S. designed the study and performed experiments; X.Z. conducted the MS experiments; B.L., J.W., and Q.L. provided clinical TNBC samples and supervised the in vivo assays performed by Y.Y., N.Y., Wei.Z., Wenlong.Z. and H.S.; W.G.Zhu, and J.J. designed the experiments and modified the manuscript. All authors critically reviewed the article and approved the final manuscript.

## Supporting information

Supporting Information
