# Supplementary material for: CDK5 Inhibition Abrogates TNBC Stem‐Cell Property and Enhances Anti‐PD‐1 Therapy
Source: Adv Sci (Weinh). 2020 Oct 15;7(22):2001417. doi: 10.1002/advs.202001417 (PMC7675186; doi:10.1002/advs.202001417)
Supplement: Supplementary file 1 — Supporting Information [file ADVS-7-2001417-s001.pdf]

WILEY-VCH

© 2020 Wiley-VCH GmbH

ADVANCED SCIENCE
Open Access

Supporting Information

for *Adv. Sci.*, DOI: 10.1002/advs.202001417

CDK5 Inhibition Abrogates TNBC Stem-cell Property and Enhances Anti-PD-1 Therapy

Yuncheng Bei, Nan Cheng, Ting Chen, Yuxin Shu, Ye Yang, Nanfei Yang, Xinyu Zhou, Baorui Liu, Jia Wei, Qin Liu, Wei Zheng, Wenlong Zhang, Huifang Su, Weiguo Zhu, Jianguo Ji,* Pingping Shen,**

WILEY-VCH

© 2020 Wiley-VCH GmbH

Supporting Information

CDK5 inhibition abrogates TNBC stem-cell property and enhances ANTI-PD-1 therapy

Yuncheng Bei, Nan Cheng, Ting Chen, Yuxin Shu, Ye Yang, Nanfei Yang, Xinyu Zhou, Baorui Liu, Jia Wei, Qin Liu, Wei Zheng, Wenlong Zhang, Huifang Su, Weiguo Zhu, Jianguo Ji,* Pingping Shen,**

Supporting information:

Title: CDK5 Inhibition Abrogates TNBC Stem-cell Property and Enhances Anti-PD-1 Therapy

Yuncheng Bei, Nan Cheng, Ting Chen, Yuxin Shu, Ye Yang, Nanfei Yang, Xinyu Zhou, Baorui Liu, Jia Wei, Qin Liu, Wei Zheng, Wenlong Zhang, Huifang Su, Weiguo Zhu, Jianguo Ji,* Pingping Shen,**

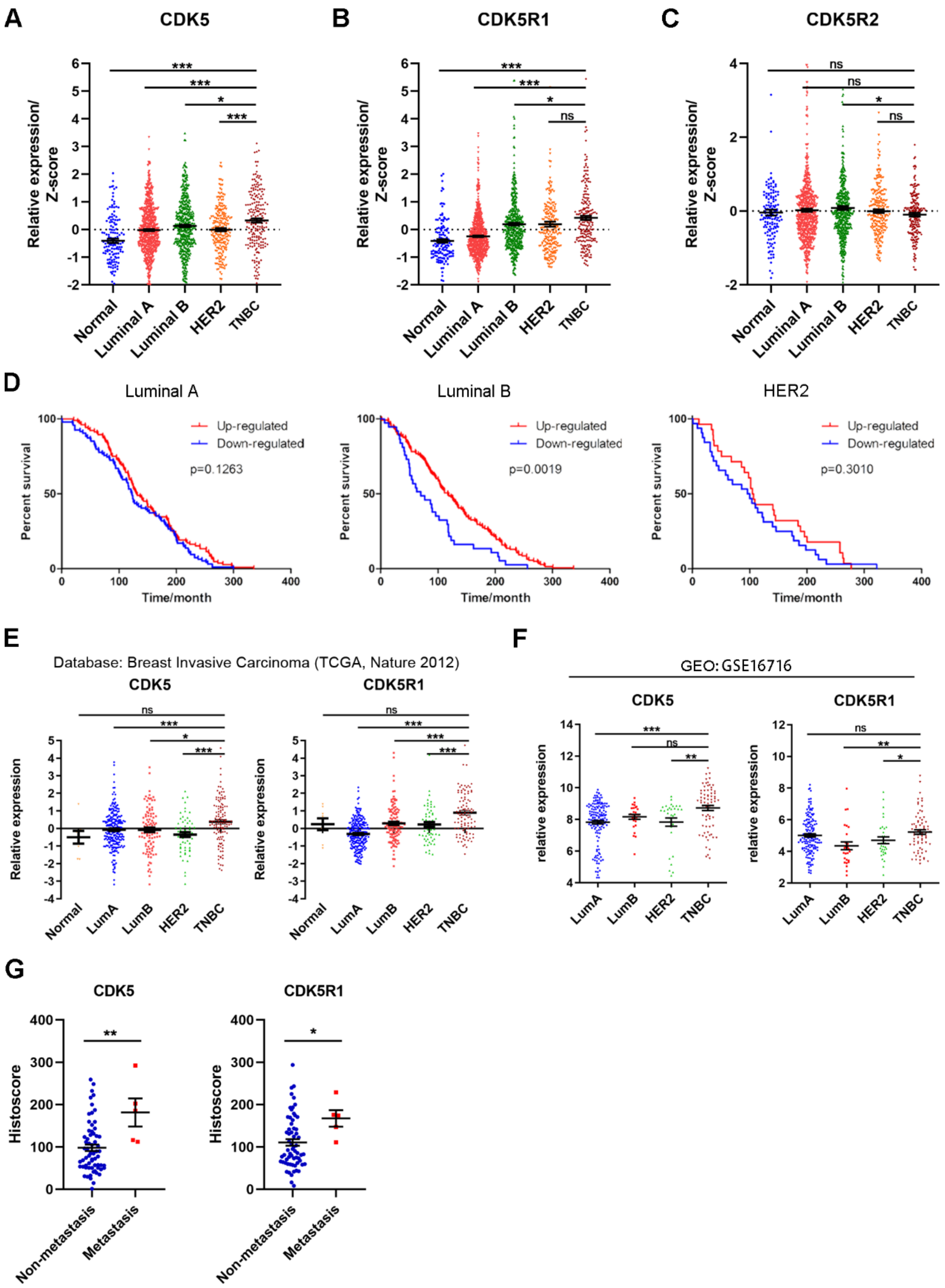

Figure S1. Aberrated expression of CDK5 and CDK5R1 in TNBC patients. A-C) TCGA database analysis available from cBioPortal for CDK5, CDK5R1 and CDK5R2 expressions in human BC database. Relative mRNA expressions of CDK5 (A), CDK5R1 (B) and CDK5R2 (C) in different subtypes of BC, including Normal (n=140), Luminal A (n=679), Luminal B (n=461), HER2 (n=220) and TNBC (n=299). D) Online analysis of survival in different subtypes of BC with high or low expression of CDK5. E, F) Relative mRNA expressions of CDK5 and CDK5R1 in different cohorts of BC as indicated. G) Histoscores for CDK5 and CDK5R1 staining in

non-metastatic and metastatic specimens. Data represent the analysis of n patient specimens per group, mean ± SEM. $p < 0.05$, *; $p < 0.01$ **; $p < 0.001$, ***.

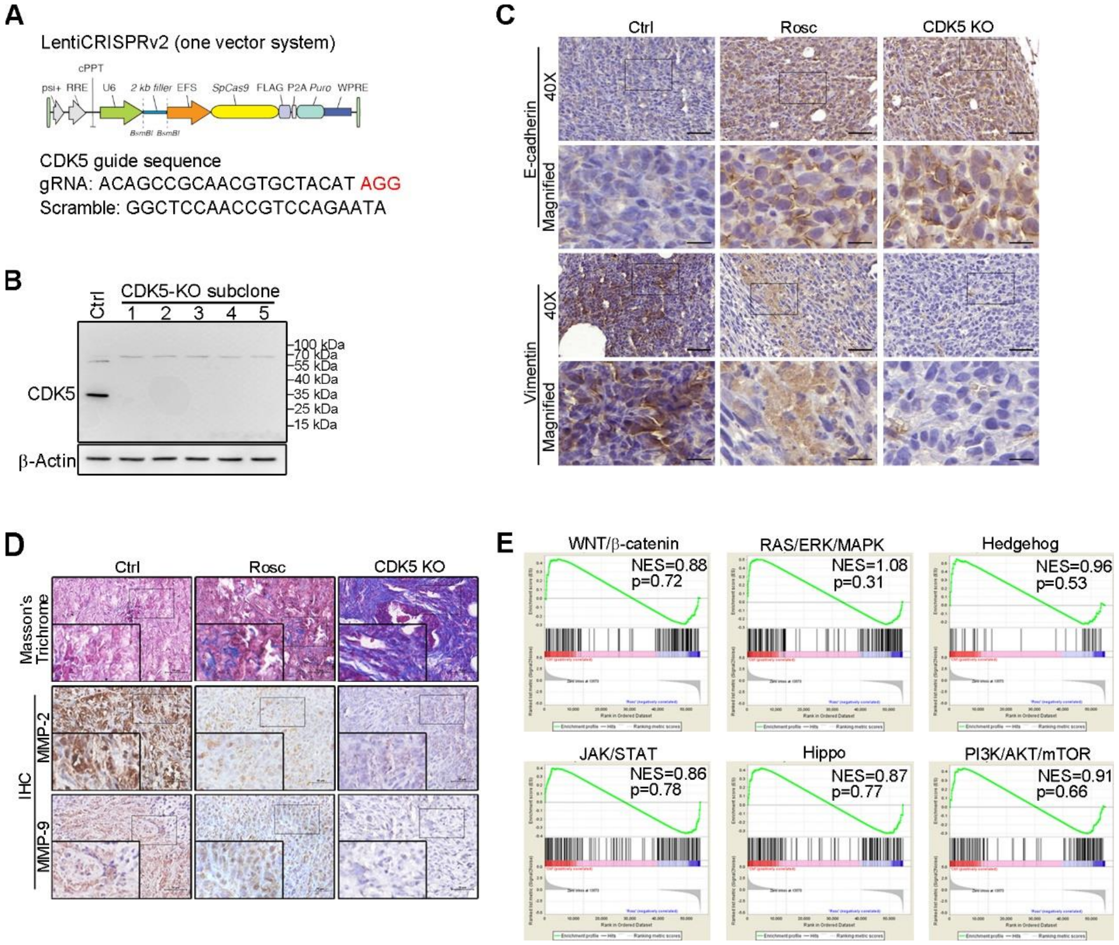

Figure S2. CDK5 interruption attenuates EMT and stemness transformation in 4T1 tumors. A) Schematic diagram of LentiCRISPRv2 (one vector system), and sgRNA for CDK5. B) Western blot analysis of CDK5 protein level in 4T1 cells with CRISPR knockout of CDK5 or parental cells. C) Representative IHC for expression of EMT markers (E-cadherin and Vimentin) in indicated tumor tissues. Scale bars, 50 μm for 40X and 20 μm for magnified field. D) Representative pictures for detection of TME remodeling from 4T1-bearing mice with different treatments as indicated. Collagen deposition was examined by Masson's trichrome and MMP-2/MMP-9 expressions were evaluated by IHC. Scale bars, 50 μm. E) Gene set enrichment analysis (GSEA) showing the enrichment of stem cell-related gene sets (as indicated) in Rosc-treated cells versus vehicle control.

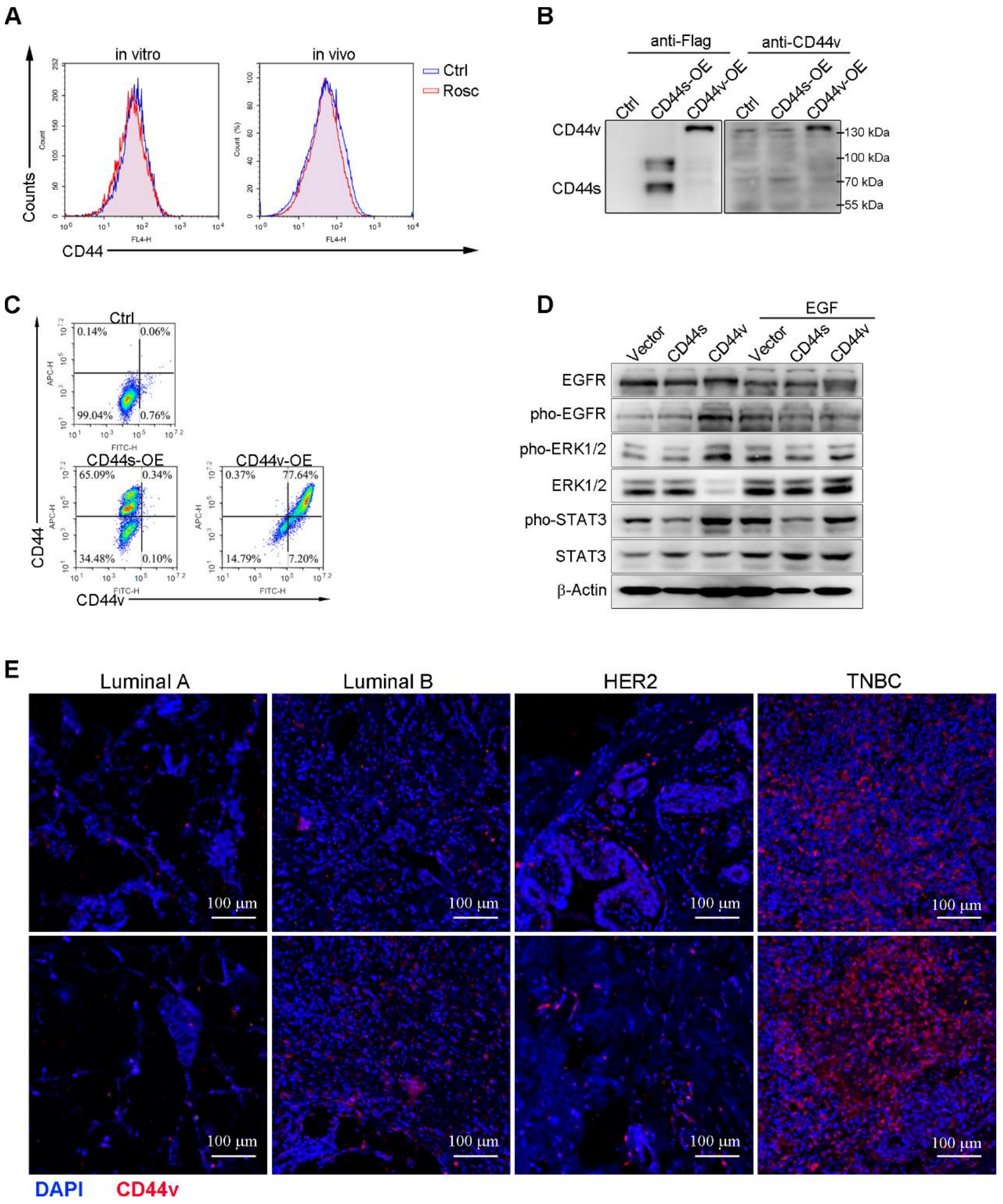

Figure S3. CD44v antibody generation and examination. A) Flow cytometric analysis of CD44 expression in vitro and in vivo. B, C) immunoblotting analysis (B) and flow cytometric analysis (C) examined the specificity of CD44v antibody by using CD44s-OE and CD44v-OE cells, respectively. D) Immunoblotting analyzed the activation of CD44v downstream EGFR signal pathway. E) Representative immunofluorescence microscopy images for analysis CD44v expression by using generated antibody in different subtypes of BC.

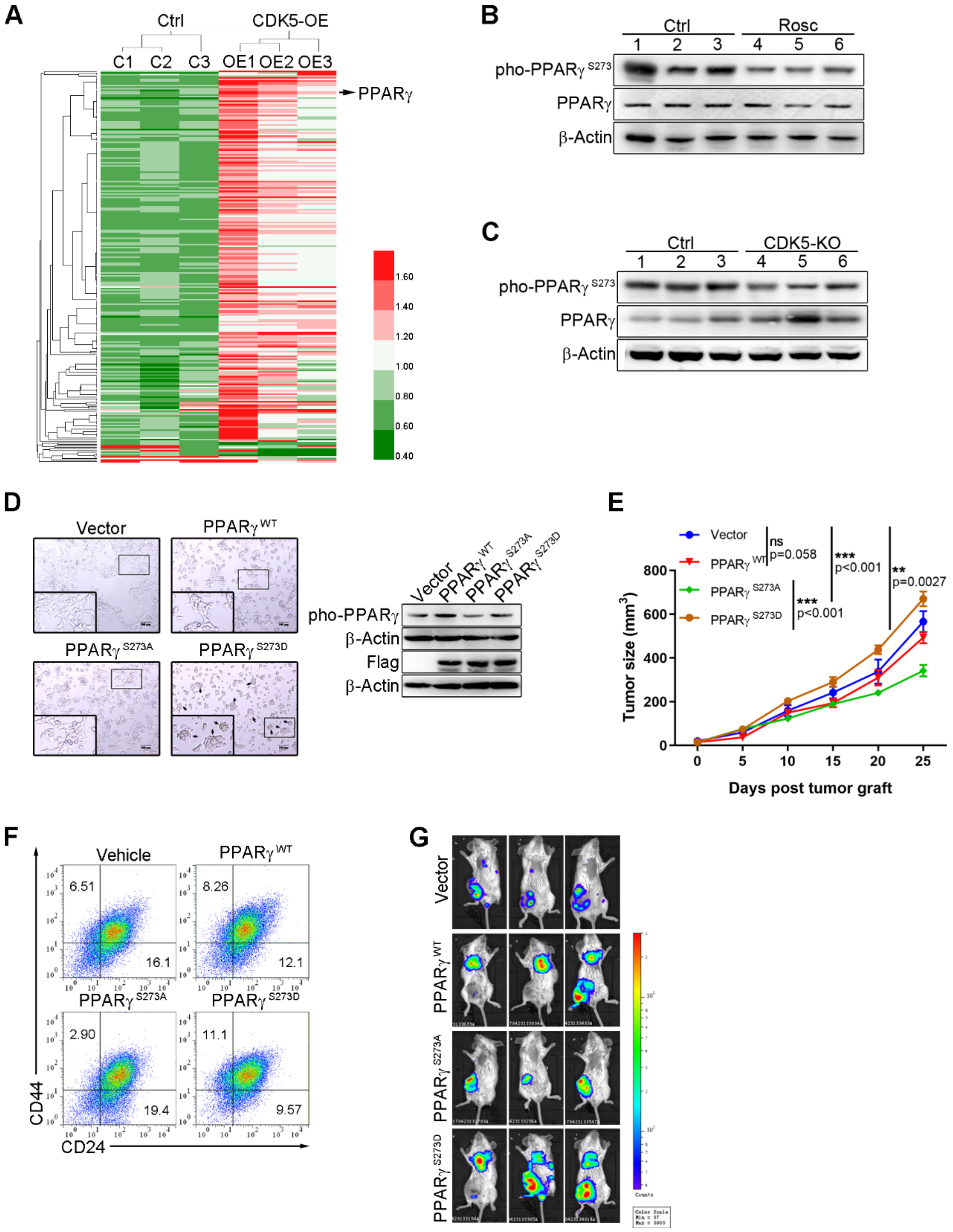

Figure S4. PPARγ phosphorylation is necessary for CDK5-induced CD44v class switching. A) Heat map of the enriched genes identified by phosphoproteome in 4T1 cells after CDK5 overexpression. PPARγ was among them (arrowhead). B, C) PPARγ was verified as downstream substrate of CDK5-mediated phosphorylation. Immunoblotting examined pho-PPARγ at Ser273 site and PPARγ expression in 4T1 cells after CDK5 interruption (B, Rosc treatment; C, CDK5 knockout). D) Establishment of wildtype PPARγ and mutated PPARγ (S273A and S273D, respectively) overexpressing 4T1 cells. immunoblotting detected PPARγ and pho-PPARγ expression in these transgenic cells. E) Establishment of orthotopically 4T1-bearing mice by using the transgenic cells. Mean tumor volumes were recorded on

indicated time. F) Flow cytometric analysis of the population of CD44^{high}CD24^{low} BCSCs in formed tumors (as indicated in E). G) Representative Bioluminescence images analysis of pulmonary metastasis in formed tumors (as indicated in E).

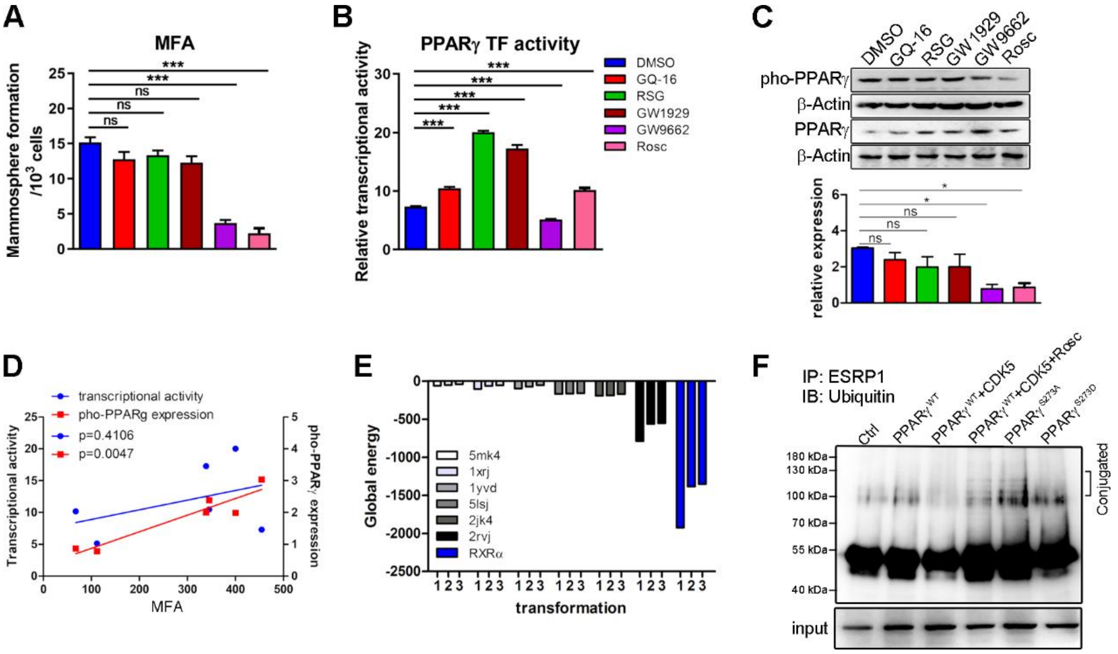

Figure S5. Pho-PPARγ induced TNBC cell stemness transformation independent on transcriptional activity. A-C) Small molecules as indicated were used to interrupt CDK5/pho-PPARγ axis in 4T1 cells. The self-renewal capacity was examined by MFA (A). The transcriptional activity was examined by dual-luciferase reporter assay (B). The pho-PPARγ expression was examined by immunoblotting (C). D) Scatter plot showing the correlation between invasive capacity of 4T1 cells treated with different PPARγ. E) Representative histograms of global energy of interaction between PPARγ and candidate proteins (PDB numbers as indicated) by online computational analysis tools (ZDock, PatchDock). Top 3 transformations of each group were listed. F) 4T1 cells were transfected with vectors expressing wild type PPARγ (PPARγ WT), mutated PPARγ (PPARγ S273A and PPARγ S273D) and CDK5R1 (p35), and treated with Rosc (as indicated). Engineered cells were pre-treated with MG132 (10 μM) for 2 hr. Cell lysates were immunoprecipitated (IP) with anti-ESRP1 and analyzed for ubiquitination with anti-ubiquitin antibody.

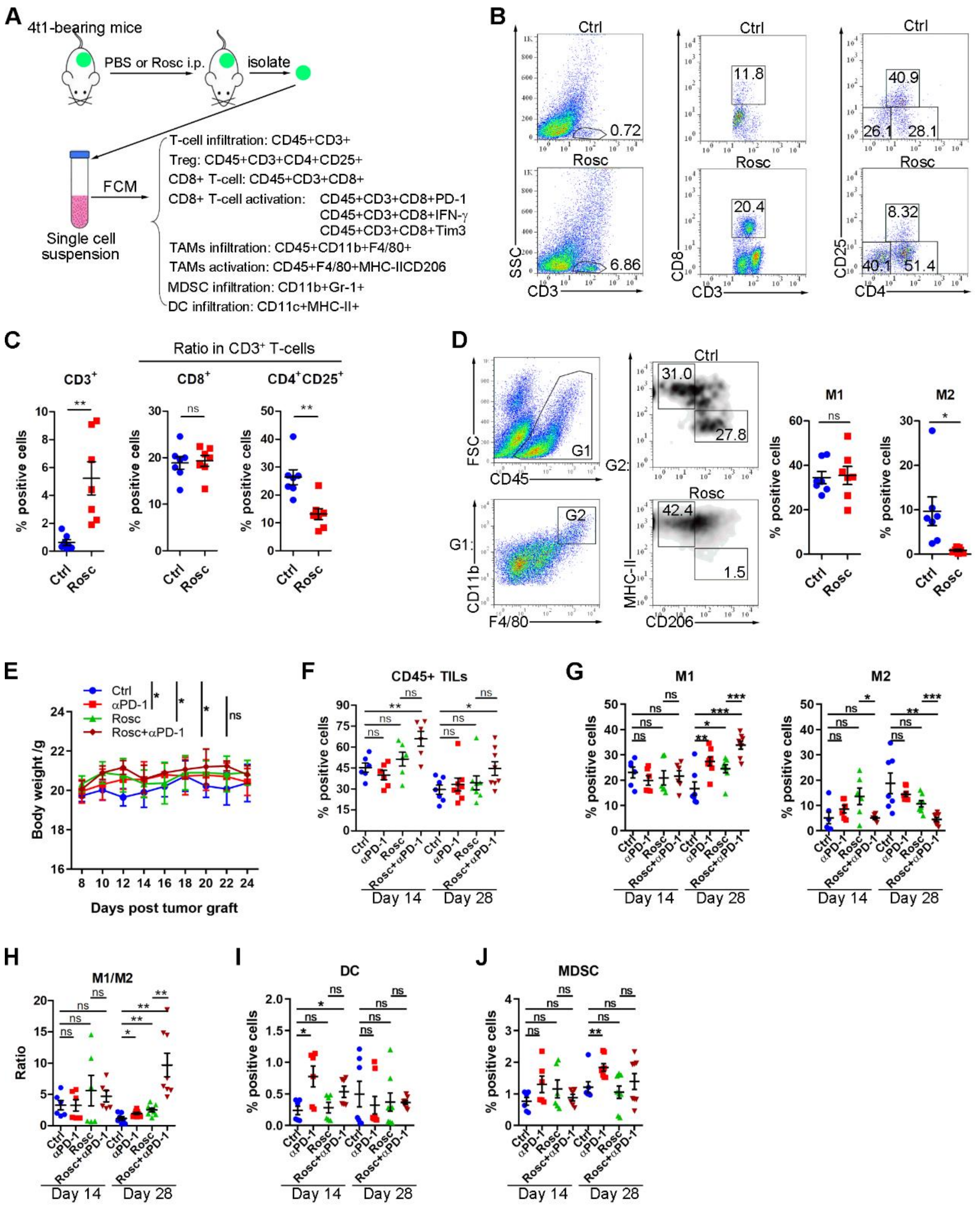

Figure S6. CDK5/pho-PPARγ axis modulates immune milieu. A) Schematic diagram of gating strategy for flow cytometric analysis of immune cells as indicated. 4T1-bearing mice were treated with Rosc versus vehicle control. Tumor tissues from tumor-bearing mice with different treatments were used to perform single cell suspension. Then single cell suspension was subjected to flow cytometric analysis. Gating strategy was shown as indicated (n=7). B-D) Representative flow cytometric analysis of whole T-cells, CTLs, Tregs, M1-prone TAMs and M2-prone TAMs population in tumors after Rosc treatment. E) 4T1-bearing mice were treated with Rosc and anti-PD-1, singly or combinedly. Body weights were recorded on indicated time. F-J) Quantification by flow cytometry of CD45+ TILs (F), M1-prone/M2-prone TAMs (G),

dendritic cells (I) and MDSC (J) population and the ratio of M1/M2 (H) in 4T1 tumors at day 14 and 28 post tumor graft (n=6).

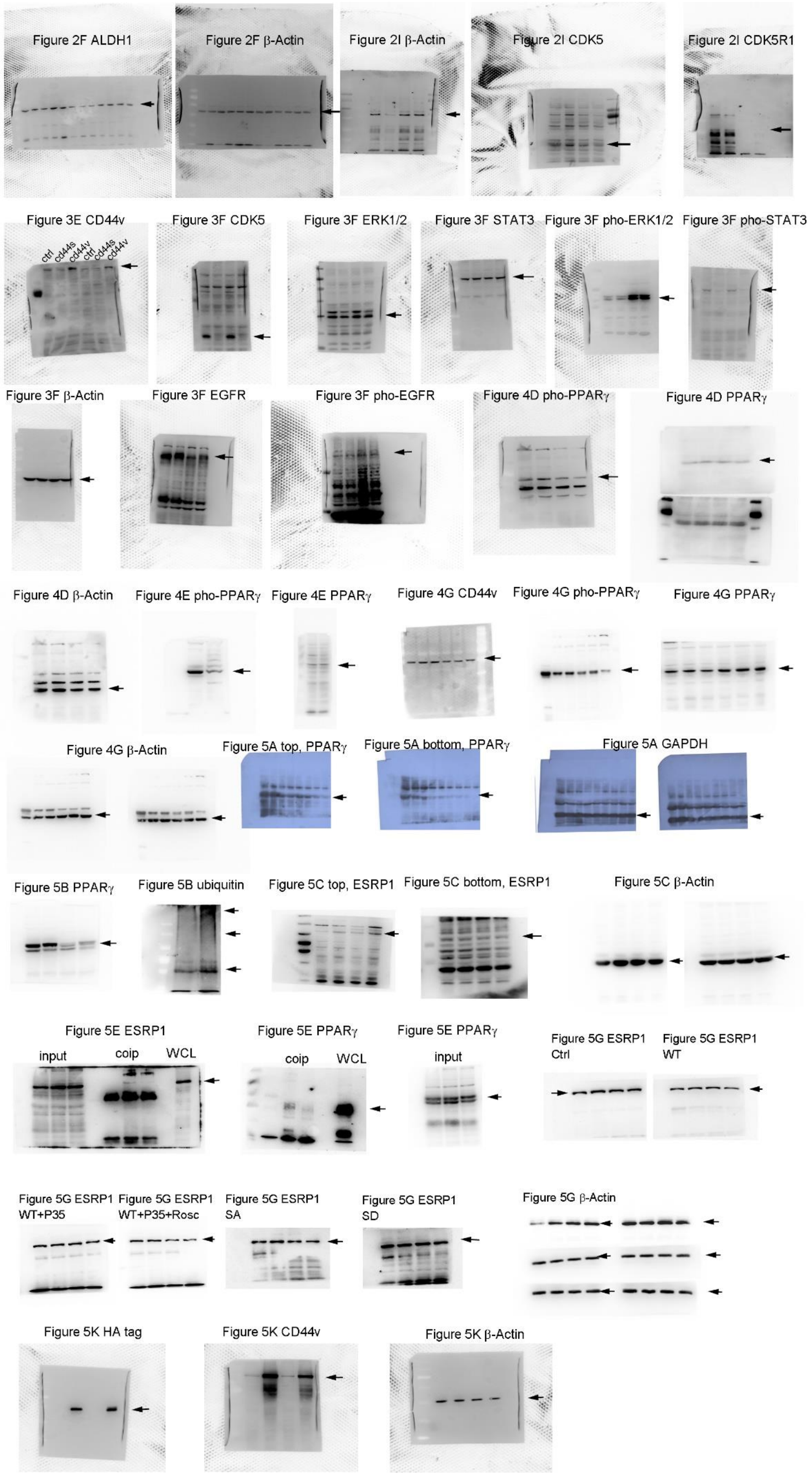
Figure 2F ALDH1
Figure 2F β-Actin
Figure 2I β-Actin
Figure 2I CDK5
Figure 2I CDK5R1
Figure 3E CD44v
ctrl cd44s cd44v ctrl cd44s cd44v
Figure 3F CDK5
Figure 3F ERK1/2
Figure 3F STAT3
Figure 3F pho-ERK1/2
Figure 3F pho-STAT3
Figure 3F β-Actin
Figure 3F EGFR
Figure 3F pho-EGFR
Figure 4D pho-PPARγ
Figure 4D PPARγ
Figure 4D β-Actin
Figure 4E pho-PPARγ
Figure 4E PPARγ
Figure 4G CD44v
Figure 4G pho-PPARγ
Figure 4G PPARγ
Figure 4G β-Actin
Figure 5A top, PPARγ
Figure 5A bottom, PPARγ
Figure 5A GAPDH
Figure 5B PPARγ
Figure 5B ubiquitin
Figure 5C top, ESRP1
Figure 5C bottom, ESRP1
Figure 5C β-Actin
Figure 5E ESRP1
input
coip
WCL
Figure 5E PPARγ
coip
WCL
Figure 5E PPARγ
input
Figure 5G ESRP1 Ctrl
Figure 5G ESRP1 WT
Figure 5G ESRP1 WT+P35
Figure 5G ESRP1 WT+P35+Rosc
Figure 5G ESRP1 SA
Figure 5G ESRP1 SD
Figure 5G β-Actin
Figure 5K HA tag
Figure 5K CD44v
Figure 5K β-Actin

Figure S7. Full scans of western blots in Figure 2F, 2I, 3E, 3F, 4D, 4E, 4G, 5A, 5B, 5C, 5E, 5K and 5G.

Table S1: Primers

b-Actin
Forward GGCTGTATTCCCCTCCATCG
Reverse CCAGTTGGTAACAATGCCATGT
ALDH1
Forward ATACTTGTCGGATTTAGGAGGCT
Reverse GGGCCTATCTTCCAAATGAACA
FGFR1
Forward TAATACCACCGACAAGGAAATGG
Reverse TGATGGGAGAGTCCGATAGAGT
NOTCH1
Forward GATGGCCTCAATGGGTACAAG
Reverse TCGTTGTTGTTGATGTCACAGT
4-Oct
Forward GGCTTCAGACTTCGCCTCC
Reverse AACCTGAGGTCCACAGTATGC
SOX1
Forward AAGGAACACCCGGATTACAAGT
Reverse GTTAGCCCAGCCGTTGACAT
ESRP1
Forward CAAGCTGGGTTCGGATGAGAA
Reverse AGGTTTTCGGCGTCTATTTTAGT
ARG-1
Forward CTCCAAGCCAAAGTCCTTAGAG
Reverse AGGAGCTGTCATTAGGGACATC
EGF
Forward AGCATCTCTCGGATTGACCCA
Reverse CCTGTCCCGTTAAGGAAAACTCT
IL-10
Forward GCTCTTACTGACTGGCATGAG
Reverse CGCAGCTCTAGGAGCATGTG
PD-L1
Forward GCTCCAAAGGACTTGTACGTG
Reverse TGATCTGAAGGGCAGCATTTC
TGF-b
Forward CTCCCGTGGCTTCTAGTGC
Reverse GCCTTAGTTTGGACAGGATCTG
VEGF
Forward CTGCCGTCCGATTGAGACC
Reverse CCCCTCCTTGTACCACTGTC

MMP-2
Forward CAAGTTCCCCGGCGATGTC
Reverse TTCTGGTCAAGGTCACCTGTC
MMP-9
Forward CTGGACAGCCAGACACTAAAG
Reverse CTCGCGGCAAGTCTTCAGAG
IFN-g
Forward ATGAACGCTACACACTGCATC
Reverse CCATCCTTTTGCCAGTTCCTC
IL-12
Forward TGGTTTGCCATCGTTTTGCTG
Reverse ACAGGTGAGGTTCACTGTTTCT
IL-1b
Forward GCAACTGTTCCTGAACTCAACT
Reverse ATCTTTTGGGGTCCGTCAACT
IL-23
Forward ATGCTGGATTGCAGAGCAGTA
Reverse ACGGGGCACATTATTTTTAGTCT
IL-6
Forward TAGTCCTTCCTACCCCAATTTCC
Reverse TTGGTCCTTAGCCACTCCTTC
iNOS
Forward GTTCTCAGCCCAACAATACAAGA
Reverse GTGGACGGGTCGATGTCAC
TNF-a
Forward CCCTCACACTCAGATCATCTTCT
Reverse GCTACGACGTGGGCTACAG
E-cadherin
Forward CAGGTCTCCTCATGGCTTTGC
Reverse CTTCCGAAAAGAAGGCTGTCC
N-cadherin
Forward AGCGCAGTCTTACCGAAGG
Reverse TCGCTGCTTTCATACTGAACTTT
Vimentin
Forward CGTCCACACGCACCTACAG
Reverse GGGGGATGAGGAATAGAGGCT
Fibronectin
Forward ATGTGGACCCCTCCTGATAGT
Reverse GCCCAGTGATTTCAGCAAAGG
Slug
Forward TGGTCAAGAAACATTTCAACGCC
Reverse GGTGAGGATCTCTGGTTTTGGTA
Snail

Forward CACACGCTGCCTTGTGTCT
Reverse GGTCAGCAAAAGCACGGTT
Twist1
Forward GGACAAGCTGAGCAAGATTCA
Reverse CGGAGAAGGCGTAGCTGAG

Table S2: antibodies

Antibodies		
Anti-CDK5	Bioworld	BS3258
Anti-CDK5R1/P35	Bioss	bs-11611R
Anti-phospho-CDK5 (Ser273)	Santa Cruz Biotechnology	sc-12919
Anti-PPARg	Cell Signaling Technology	2443S
Anti-PPARg (E-8)	Santa Cruz Biotechnology	sc-7273
Anti-pho-PPARγgSer273)	Bioss	bs-4888R
Anti-ERK1/2	Bioworld	BS1112
Anti-phospho-ERK1/2 (T202/Y204)	Bioworld	BS5016
Anti-ALDH1	Bioss	bs-10162R
Anti-Ki-67	Cell signaling technology	#2586
Anti-E-cadherin	Santa Cruz Biotechnology	sc-7870
Anti-Vimentin	Bioss	bsm-33170M
Anti-ESRP1	Bioss	bs-19771R
Anti-Ubiquitin	Bioworld	BS1487
Anti-CD44v6 mouse	Bioss	bs-20756R
Anti-b-Actin-HRP conjugated	Abclonal	AC028
Anti-GAPDH-HRP conjugated	KANG CHEN	KC-5G5
Anti-FLAG	Bioworld	AP0007
Anti-PD-1	Bioxcell	BE0146
Rat IgG2a isotype control	Bioxcell	BE0089
Anti-CD11b PE-conjugated	eBioscience	11-0112
Anti-CD11c PE-conjugated	eBioscience	45-0114
Anti-CD206 PE-conjugated	Biolegend	141705
Anti-CD24 PE-conjugated	BD Biosciences	553262
Anti-CD25 PerCP-Cyanine5.5-conjugated	Biolegend	101911
Anti-CD3e PE-conjugated	eBioscience	12-0031
Anti-CD4 FITC-conjugated	eBioscience	11-0041
Anti-CD44 APC-Cy7-conjugated	Biolegend	103027
Anti-CD45 FITC-conjugated	eBioscience	11-0451
Anti-CD45 PerCP-Cyanine5.5-conjugated	eBioscience	45-0451
Anti-CD8a PerCP-Cyanine5.5-conjugated	eBioscience	45-0081
Anti-F4/80 FITC-conjugated	eBioscience	11-4801

Anti-F4/80 APC-conjugated	eBioscience	17-4801
Anti-Gr1 PE-conjugated	eBioscience	12-5931
Anti-IFN-g APC-conjugated	eBioscience	17-7311
Anti-MHC-II (I-A/I-E) FITC-conjugated	eBioscience	11-5321
Anti-PD-1 APC-conjugated	eBioscience	17-9981
Anti-PD-L1 PE-conjugated	BD Biosciences	558091
Anti-TIM3 APC-conjugated	eBioscience	17-5871
